# Factors influencing implementation of an Alzheimer’s disease blood test among UK old age psychiatrists: mixed-methods study using the theoretical domains framework

**DOI:** 10.1093/ageing/afag117

**Published:** 2026-05-06

**Authors:** Jemma Hazan, Mitchell Mealing, Fabiana Lorencatto, Penny Rapaport, Ashvini Keshavan, Jonathan M Schott, Joanne Rodda, Robert Howard

**Affiliations:** Division of Psychiatry, University College London, London, UK; North London NHS Foundation Trust, London, UK; North London NHS Foundation Trust, London, UK; Centre for Behaviour Change, Department of Clinical, Educational and Health Psychology, University College London, London, UK; Division of Psychiatry, University College London, London, UK; North London NHS Foundation Trust, London, UK; Dementia Research Centre, UCL Queen Square Institute of Neurology, London, UK; Dementia Research Centre, UCL Institute of Neurology, London, London, UK; UK Dementia Research Institute at UCL, London, UK; Kent and Medway Medical School, Canterbury, UK; Kent and Medway NHS and Social Care Partnership Trust, Maidstone, UK; Division of Psychiatry, University College London, London, UK; North London NHS Foundation Trust, London, UK

**Keywords:** Alzheimer’s disease, dementia, blood biomarker, behaviour change, qualitative research, older people

## Abstract

**Introduction:**

Only 6% of UK memory services meet Alzheimer’s disease (AD) biomarker access guidelines, limiting psychiatrists’ experience. Emerging AD blood biomarker (BBM) tests will potentially expand access. Exploring implementation barriers and enablers a priori can inform rollout strategies. This study examined current clinical practises, barriers and enablers to implementation and potential interventions to support implementation.

**Methods:**

In November 2024, Royal College of Psychiatrists Old Age Psychiatry Faculty members (*n* = 172) participated in an online survey and four focus groups (*n* = 16 participants), informed by the Theoretical Domains Framework (TDF) and Behaviour Change Wheel. Demographic data were summarised descriptively. Mean (SD) belief statement scores for TDF domains and percentage agreement were calculated. Multiple linear regression examined associations between TDF domains and intention to use BBMs.

**Results:**

Respondents were mainly consultants in England; <33% had used biomarkers. Key barriers to use were: ‘Knowledge,’ ‘Environmental Context and Resources’ and ‘Goals.’ Enablers included: ‘Behavioural Regulation,’ ‘Social Influences’ and ‘Intention.’ Mixed enablers/barriers included: ‘Beliefs about Consequences’, ‘Optimism’ and ‘Social/Professional Role & Identity’. In regression analyses, ‘Memory, Attention and Decision Processes’ (B = 0.44, 95%CI 0.20–0.68), ‘Beliefs about Consequences’(B = 0.45, 95%CI 0.11–0.78), and ‘Social Influences’ (B = 0.24, 95%CI 0.04–0.44) were positively associated with intention, while ‘Optimism’ (B = -0.31,95% CI-0.58 to-0.04) and ‘Emotion’ (B = -0.33, 95%CI -0.60 to-0.06) were negatively associated. Key interventions were ‘Guidelines’ (e.g. appropriate use criteria) and ‘Environmental Restructuring’ to expand resources and re-organise pathways.

**Discussion:**

A complex interplay of barriers and enablers influences AD BBM implementation. Interventions targeting clinician, service and policy levels are required to support their introduction.

## Key Points

- UK Survey respondents from the Royal College of Psychiatrists Old Age Faculty have very limited experience of Alzheimer’s disease (ad) biomarker investigations. This may more broadly reflect the experience of UK memory service psychiatrists.
- ad BBMs are rapidly advancing and likely to be implemented in clinical practise soon; however, addressing identified barriers to their use will be essential to ensure successful rollout and maximise implementation.
- Barriers include limitations in awareness of ad BBM and their appropriate use, resource constraints and pathway integration concerns. Cautious optimism was a mixed barrier/enabler, while pressure from patients or families to request testing, and intention to use were identified as enablers.
- Despite a small survey sample size, these findings could provide the basis for more targeted interventions in clinical practise and should be triangulated with other studies. Potential strategies identified were the development of appropriate use criteria, environmental restructuring and an education/training package for clinicians.

## Introduction

In the United Kingdom (UK), Alzheimer’s disease (AD) dementia is most often diagnosed in National Health Service (NHS) community memory services [1], by multidisciplinary teams, usually led by old age psychiatrists. Misdiagnosis rates of AD are around 30% when compared with post-mortem neuropathology [2]. National Institute for Health and Care Excellence (NICE) 2018 guidelines recommend biomarker testing, including cerebrospinal fluid (CSF) analysis, if the diagnosis is uncertain and Alzheimer’s disease is suspected [3]. However, only 2% of memory service patients currently have any specialised diagnostic tests (e.g. PET, DAT SPECT and CSF) [4], with notable regional disparities [5], due to resource constraints and insufficient clinician training [6, 7]. Consequently, in cases where diagnosis requires molecular diagnostic confirmation, patients are typically referred to specialist tertiary neurology centres following initial assessment [8], thus limiting psychiatrists’ experience with use of molecular diagnostic biomarkers in clinical practise.

The need for biomarker-supported AD diagnoses is growing due to the advent of potential disease-modifying treatments [9]. Advances in AD blood-based biomarker tests (BBMs) could improve access to testing within community settings [10]. However, identifying and addressing modifiable barriers and enablers to adopting AD BBMs is crucial to facilitate their future implementation into clinical practise. Conducting a behavioural analysis prior to implementation can help identify strategies that might be needed as part of their roll out to maximise and enable implementation [11, 12].

Implementing innovations in healthcare, such as adoption of a new AD BBM, inherently involves behaviour change [13]. It requires healthcare professionals (HCPs) to conduct the test, communicate the results and make consequent treatment decisions. Patients and their caregivers must consent to testing. Successful implementation can be enhanced by understanding the factors that influence human behaviour and behaviour change theories and frameworks can help us to understand implementation challenges. The Theoretical Domains Framework (TDF), widely used for this purpose [14], synthesises constructs from 33 behaviour change theories into 14 domains [13]. These domains encompass a range of influences on behaviour, including individual (e.g. knowledge and beliefs), sociocultural (e.g. professional identity) and environmental influences (e.g. resource availability and clinical context). The TDF provides a structured and systematic approach to understanding the multifaceted influences of behaviour in clinical settings. It has been widely used to structure interviews and surveys exploring various clinical practise implementation behaviours, including in the context of dementia [15].

Various studies have investigated influences on the uptake of biomarker tests [16, 17]. We conducted a qualitative evidence synthesis of these studies [18] investigating perceptions of patients, caregivers and HCPs regarding AD biomarker use through the TDF lens. This synthesis identified that key barriers to use of AD biomarkers were in the domains ‘Knowledge’ (e.g. enhancing understanding of biomarkers) and ‘Emotion’ (e.g. emotional burden following testing). Key enablers were in the domains ‘Beliefs about Consequences’ (e.g. addressing perceived benefits and risks) and ‘Memory, attention and decision processes’ (e.g. supporting shared decision making and test result communication). However, research specifically focused on UK psychiatrists’ perspectives remains limited, and existing studies are not theoretically informed [5]. Drawing on behavioural frameworks such as the TDF is therefore particularly relevant for investigating behavioural influences of AD biomarker implementation in this context.

After identifying barriers and enablers to adopting AD BBMs, the next step is to develop targeted intervention strategies to support implementation. A benefit of the TDF is that it is mapped to an integrated framework, the Behaviour Change Wheel (BCW) [19]. The BCW specifies nine broad intervention types to change behaviour: such as education, persuasion, training and environmental restructuring [20]. The BCW is mapped to the TDF to suggest which types of interventions are more likely to be effective in overcoming barriers and enhancing enablers across TDF domains. This integration enables a systematic, evidence-based approach to developing interventions that address barriers and leverage enablers [21], with the goal of promoting the adoption of BBMs.

Building on findings from our recent qualitative systematic review [18], this mixed-methods study aimed to apply behavioural science frameworks to: (i) Describe current AD biomarker practises among UK psychiatrists involved in the diagnosis of dementia; (ii) Qualitatively and quantitatively identify perceived barriers and enablers to AD BBM use and compare differences in responses based on whether the psychiatrists works in specialist memory services or alternative clinical settings; (iii) Use regression modelling to examine the associations between TDF domains and intention to use AD BBMs; and (iv) Identify potential intervention strategies using the BCW that would support the implementation of AD BBMs.

## Methods

### Design

Theory-based, cross-sectional online survey and focus groups were conducted. UCL Ethics Committee approval was gained (reference 27,287/001).

### Cross-sectional survey

#### Participants and recruitment strategy

The survey was disseminated via an email invitation in November 2024 to all members of the Faculty of Old Age Psychiatry at the Royal College of Psychiatrists (*n* = 5269).

#### Questionnaire

The survey was created based on guidelines for conducting surveys using the TDF [13]. The full survey is available in Appendix S1 in the Supplementary Data. It was designed to ensure complete anonymity and is structured into five sections as shown in Table 1.

**Table 1:** Data points collected in each section of the survey

Section	Data points collected
**1—Participant demographics**	Years of experience in Old Age Psychiatry, training in other specialties, current job position, geographic region in the UK, whether they worked in an academic centre and details of their current service setting.
**2—Current clinical practise**	Number of patients evaluated for ad, the proportion of their practise involving ad patients, types of investigations used in routine assessments, the percentage of patients for whom amyloid biomarker investigations were ordered, reasons for ordering such investigations and the percentage of patients referred to neurology for further diagnostic evaluation.
**3—Barriers and enablers to AD BBM use**	5-point Likert scoring spanning Strongly Agree to Strongly Disagree for 49 statements mapped to 13 TDF domains covering barriers and enablers to the use of AD BBMs. The survey, annotated with the corresponding TDF domains for each statement, is provided in the Appendix Free text question—‘Is there anything else you see as a challenge or an opportunity in using a blood biomarker test for Alzheimer’s disease?’
**4—Intention to use AD BBMs**	7-point Likert scoring spanning Strongly Agree to Strongly Disagree for 3 belief statements addressing participants’ intentions to use AD BBMs.
**5—Intervention strategies**	5-point Likert scoring spanning Strongly Agree to Strongly Disagree for 18 statements reflecting potential intervention strategies aligned with BCW intervention functions. The survey section corresponding to these items is annotated in the Appendix, indicating the BCW intervention functions linked to each question. Free text question inviting participants to provide any additional comments not covered in the survey.

Abbreviations: AD, Alzheimer’s disease; BBM, blood biomarker; BCW, behaviour change wheel; TDF, theoretical domains framework; UK, United Kingdom.

A draft questionnaire was piloted among three old age psychiatrists to test the clarity and face validity of questions and participant burden and revised accordingly.

#### Procedure

The survey was conducted anonymously using Qualtrics survey software [22], with informed consent obtained by completion of a pre-survey consent form. Participants could withdraw at any time without reason and could skip optional open-text responses. All other questions required a response to proceed. Participants were entered into a draw to win one of eight £100 vouchers.

#### Analyses

The quantitative component aimed to characterise beliefs regarding barriers and enablers to AD BBM implementation, examine differences by clinical setting and identify TDF domains associated with intention to use AD BBMs.

Analyses were performed using R version 4.1.2 [23] using complete responses. Demographics and current practise responses (e.g. diagnosis rates) were summarised with descriptively (n,%). Belief statements were mapped to 13 TDF domains. The mean score and standard deviation (SD) were calculated across respondents for each belief statement for descriptive purposes (Appendix S2), along with percentage agreement scores (proportion of respondents selecting ‘Agree’ or ‘Strongly Agree’). Negatively framed TDF belief statements were reverse-coded so higher scores reflected more positive attitudes.

Differences in percentage agreement between memory services and other settings, academic and non-academic centres were assessed using Fisher’s exact test with Bonferroni correction.

An unadjusted multiple linear regression model examined associations between domain scores and intention. For each TDF domain, a composite (scale) score was calculated for each participant by averaging the item responses. ‘Intentions’ was not included as a domain within the barrier and enabler questions and was instead used as a proximal outcome variable [24]. This was measured as the mean score of three 7-point Likert-scale items (−3 to +3), with higher scores indicating stronger intention to use AD BBMs. This allowed us to explore its association with TDF domains. The limited current roll-out of AD BBMs meant that measuring actual behaviour (i.e. current practise) was not feasible. Regression coefficients (B) with 95% confidence intervals (CIs) were reported. Sensitivity analyses were conducted including consultant role and geographic region as covariates. Model assumptions were tested for linearity, homoscedasticity, normality, autocorrelation and multicollinearity.

Two investigators (JH, MM) independently coded open-text survey responses in NVivo12. Similar responses for barriers/enablers (survey section 3) and interventions (survey section 5) were grouped, and theme labels inductively generated. Themes related to barriers and enablers were deductively mapped to TDF domains and themes related to potential intervention suggestions were deductively coded to BCW intervention types. The research team reviewed themes and classifications.

### Focus groups

#### Participants and sampling

Focus group participants were recruited using self-selected purposive sampling from the survey sample. In the survey, respondents were asked to tick a box if they were willing to be contacted for participation in focus groups to explore the topic in depth. Data collection continued until thematic saturation was achieved, defined as the point at which no new codes or concepts emerged from successive transcripts and the coding framework had stabilised. [25] Saturation was monitored iteratively by the research team through concurrent data collection and analysis, with each new focus group transcript reviewed for novel themes.

#### Procedure

Focus groups were conducted via videocall by JH, PR & RH. All participants provided consent before participating in the focus groups. The focus groups were audio-recorded, transcribed verbatim and fully anonymised.

#### Topic guide

The semi-structured focus group topic guide (Appendix S3) was developed by the research team and included open-ended questions to examine barriers and enablers to use of an AD BBM in greater depth. These questions were informed by key TDF domains identified through findings from our systematic review [18] and survey, with additional questions exploring potential intervention strategies. The topic guide was piloted and refined prior to data collection.

#### Data analysis

Transcripts were independently analysed by two researchers (JH & MM) using NVivo12 and Microsoft Word, following guidelines for applying the TDF in data analysis [13], using a combined deductive framework approach with inductive thematic analysis. Further information regarding the thematic analysis is available (Appendix S4). Survey and focus group findings were then integrated through narrative triangulation, with the TDF domains serving as a common analytical framework to compare quantitative and qualitative data and enhance the interpretation of key barriers and enablers.

## Results

The results from the survey and focus groups are presented and discussed together below.

### Demographic characteristics

One hundred and seventy-two complete survey responses were received (from 242 total responses, dropout rate 28.9%). Respondent demographic characteristics are presented in Table 2 and Appendix S5. The majority were consultant psychiatrists (63%) and based in England (84%). Around one-third had trained in a different specialty (34%), half worked in academic centres (50%) and almost two-thirds worked in memory services (62%).

**Table 2:** Key demographic details of survey participants

Demographics		*n*	% of total
Years practising Old Age Psychiatry	1–10	72	42
	11–20	64	37
	20+	36	21
Role	Consultant	109	63
	All other grades	63	37
Trained in other specialty	Yes	58	34
	No	114	66
Region	England	144	84
	Northern Ireland	3	2
	Scotland	18	11
	Wales	7	4
Works in Academic Centre	Yes	86	50
	No	86	50
Service	Memory service	107	62
	All other	65	38

*Definitions: Role (all other grades)* includes Specialty Trainee, Core Trainee and Specialty and Associate Specialist (SAS) Doctor. *Setting (all other settings)* includes Inpatient setting, Community Mental Health Team (CMHT), Liaison Service and Other.

Sixteen psychiatrists in 4 focus groups (December 2024–January 2025) explored AD BBM implementation; 69% were consultants, 56% had 1–5 years of experience in old age psychiatry and 63% worked in memory services (Appendix S6).

### Current practise

The majority of survey respondents reported making an AD diagnosis at least once a week (62.2%). The most frequently used investigations were CT imaging (82.6%), MRI imaging (69.2%) and neuropsychology assessments (48.3%). Less than a third of respondents reported ordering biomarker investigations for their patients (32.6%), with the majority of these doing so for atypical (78.5%) or young onset (79.1%) presentations. Additionally, 82.0% of respondents referred fewer than 5% of their patients to a neurology centre for further assessment (Appendix S7).

In the focus groups, HCPs expanded on this, explaining that they did not have access to biomarker testing locally *‘very few people, you know, including myself have really had a lot of access to it’* (Participant 7, Consultant) and would need to refer to neurology *‘we don’t have access to those more sophisticated tests…except by way of referral to neurology’* (Participant 13, Consultant). Discourse over when they may use biomarker investigations included agreement with NICE guidelines *‘you’d use them currently when a person’s got dementia and unsure what the cause or causes are and will it affect outcome not using on its own in MCI [mild cognitive impairment]’.* (Participant 5, Consultant).

### Barriers and enablers to AD BBM use

Table 3 presents survey results of agreement and disagreement with belief statements regarding barriers and enablers to AD BBM use, along with TDF domain definitions [26]. Analysis of open-text survey responses (*n* = 59) identified themes mapped to TDF domains, with key barriers and enablers discussed within each domain below and full details of themes and sub-themes in Appendix S8.

**Table 3:** Mean scores and percentage agreement with belief statements representing barriers and enablers to AD BBM use

Belief statement (corresponding TDF domain)	Mean scores		% agreement
	Mean	SD	All
**Knowledge** (An awareness of the existence of something)			
I am familiar with the evidence base supporting the use of blood biomarkers for Alzheimer’s disease	3.24	1.07	47.1
I have a good understanding of what blood biomarkers for Alzheimer’s disease measure	3.41	0.94	55.2
I know the currently published appropriate context of use recommendations to request a blood biomarker for Alzheimer’s disease	2.79	1.04	26.7
**Memory, attention and decision processes** (The ability to retain information, focus selectively on aspects of the environment, and choose between two or more alternatives)			
I would bear in mind using a blood biomarker for Alzheimer’s disease as part of my routine clinical practise	3.80	0.98	71.5
Using a blood biomarker result for Alzheimer’s disease would strongly inform my clinical decision making around diagnosis	3.80	0.89	68.0
Using a blood biomarker for Alzheimer’s disease would require me to expend significantly more effort in making a decision than current practise	2.62	0.99	20.9
**Skills** (An ability or proficiency acquired through practise)			
It would require technical skill to take a blood sample for a biomarker test for Alzheimer’s disease	2.64	1.12	26.2
I feel confident I could communicate a blood biomarker for Alzheimer’s disease result to colleagues in the team	3.76	0.90	72.1
I feel confident I could communicate a blood biomarker for Alzheimer’s disease result to patients and their families	3.73	0.92	71.5
**Behavioural Regulation** (Anything aimed at managing or changing objectively observed or measured actions)			
I would want all patients presenting to our clinic for investigation of possible dementia to have this test	2.76	1.14	30.8
I would work with my colleagues in our service to develop local policy for use of the test	4.16	0.66	88.4
I would be willing to compare my use of this blood biomarker test with local colleagues	4.29	0.55	95.3
**Social/professional role and identify** (A coherent set of behaviours and displayed personal qualities of an individual in a social or work setting)			
It is my responsibility to use the latest advancements in diagnostic technology when they become available for my patients	4.08	0.83	82.0
Using a blood biomarker test for Alzheimer’s disease would align with best practise for dementia diagnosis	3.84	0.78	70.3
I have ethical concerns to using a blood biomarker test for Alzheimer’s disease in clinical practise (specifically not genetic blood tests such as APOE status)	2.41	0.94	14.5
A delay in time between MHRA (Medicines and Healthcare products Regulatory Agency) approval and NICE guidance for a blood biomarker test for Alzheimer’s disease would stop me ordering the test	3.32	0.97	48.3
Clinicians working in old age psychiatry should use a blood biomarker test to improve diagnoses in Alzheimer’s disease	3.84	0.75	70.9
**Beliefs about capabilities** (Acceptance of the truth, reality, or validity about an ability, talent, or facility that a person can put to constructive use)			
I have confidence in my ability to use a blood biomarker for Alzheimer’s disease test in my diagnostic practise	3.37	0.95	53.5
I would feel confident my allied health professional colleagues would be able to use a blood biomarker for Alzheimer’s disease test in their diagnostic practise	2.47	0.99	16.9
I feel confident that I could interpret the result of a blood biomarker for Alzheimer’s disease	3.49	0.91	57.0
**Optimism** (The confidence that things will happen for the best or that desired goals can be attained)			
I believe a blood biomarker for Alzheimer’s disease will have high accuracy for detecting Alzheimer’s pathology in the brain	3.52	0.75	52.9
I believe a blood biomarker for Alzheimer’s disease would improve how Alzheimer’s dementia is diagnosed	3.98	0.67	84.3
I do not believe a blood biomarker for Alzheimer’s disease would improve how Alzheimer’s dementia is treated	2.41	1.07	18.0
**Beliefs about consequences** (Acceptance of the truth, reality, or validity about outcomes of a behaviour in a given situation)			
I will have access to more helpful information to guide diagnosis	3.99	0.64	87.8
It may reassure the patient/family that the diagnosis is reliable	4.01	0.64	86.6
It would help me facilitate access to medication licenced for Alzheimer’s disease	3.62	1.01	65.1
It will help to establish the diagnosis of Alzheimer’s disease earlier	3.99	0.74	82.6
The test result may have indeterminant (not black and white) values	3.95	0.678	82.6
The result may conflict with a diagnosis suggested by the clinical presentation	3.85	0.68	80.2
The result may conflict with the results of other investigations (e.g. imaging)	3.70	0.74	72.1
In a busy clinic the additional time and waiting for a result associated with the blood test could prevent it’s use	2.69	1.02	26.7
**Goals** (Mental representations of outcomes or end states that an individual wants to achieve)			
My personal target is to reduce the number of patients who are diagnosed with MCI	2.99	0.98	34.3
There are targets in my service related to increasing the proportion of patients with a pathological diagnosis	2.65	1.05	23.3
It would be a priority for me to incorporate a blood biomarker test for Alzheimer’s disease in my practise, relative to currently available investigations (e.g. brain imaging)	3.44	0.99	54.7
**Reinforcement** (Increasing the probability of a response by arranging a dependent relationship, or contingency, between the response and a given stimulus)			
Having access to a blood biomarker test for Alzheimer’s disease will make me more confident in my diagnostic skill	3.70	0.86	70.3
Commissioners of services are more likely to fund our service if we are using a blood biomarker test for Alzheimer’s disease as part of our assessment protocol	3.06	0.94	27.9
I would avoid using the blood biomarker test for Alzheimer’s disease because a positive result could distress my patients	1.81	0.69	1.2
**Emotion** (A complex reaction pattern, involving experiential, behavioural and physiological elements, by which the individual attempts to deal with a personally significant matter or event)			
I feel positive about using a blood biomarker test for Alzheimer’s disease in clinical practise	3.92	0.76	76.7
I feel frustrated about having to change what I do currently in the diagnostic assessment of Alzheimer’s disease	1.85	0.72	3.5
I feel threatened that the result of a blood biomarker test for Alzheimer’s disease may replace my expertise and skills	1.78	0.79	4.1
**Environmental Context and Resources** (Any circumstance of a person’s situation or environment that discourages or encourages the development of skills and abilities, independence, social competence and adaptive behaviour)			
I have access to an appropriate space (e.g. clinic room) to use a blood biomarker test for Alzheimer’s disease in clinical practise	3.25	1.20	55.8
I have access to basic blood test equipment to use a blood biomarker test for Alzheimer’s disease in clinical practise	3.20	1.19	53.5
There are available trained staff in phlebotomy to use a blood biomarker test for Alzheimer’s disease in clinical practise	3.09	1.22	45.9
I have access to blood sample transportation to use a blood biomarker test for Alzheimer’s disease in clinical practise	3.09	1.19	45.9
I have access to a laboratory to analyse any blood samples I may request in clinical practise	3.21	1.09	48.8
I have enough time to incorporate a blood biomarker test for Alzheimer’s disease in clinical practise	3.47	0.93	57.0
**Social Influences** (Those interpersonal processes that can cause individuals to change their thoughts, feelings or behaviours)			
Most of my colleagues within my professional discipline would think that using a blood biomarker test for Alzheimer’s disease is a good idea	3.69	0.78	65.1
Most of my patients would think that using a blood biomarker test for Alzheimer’s disease is a good idea	3.87	0.74	71.5
Most of my patients’ families would think that using a blood biomarker test for Alzheimer’s disease is a good idea	3.89	0.70	75.6
**Intentions** (A conscious decision to perform a behaviour or a resolve to act in a certain way)			
I intend to use a blood biomarker test for Alzheimer’s disease in clinical practise if they are approved for use	2.05	1.03	93.0
I want to use a blood biomarker test in clinical practise if it is approved for use	2.09	1.02	93.6
I believe I will be able to use a blood biomarker test in clinical practise if it is approved for use	1.81	1.06	88.4
Mean scores reflect the extent of participant agreement with each statement. Most items used a 5-point Likert scale (1 = strongly disagree to 5 = strongly agree). For statements within the ‘Intentions’ domain, a 7-point Likert scale was used (−3 = strongly disagree to +3 = strongly agree).			

Table 4 provides a summary of identified barriers, enablers and mixed barriers/enablers per theoretical domain with example supporting quotes in the focus groups. A narrative summary of the enablers and barriers for implementing AD BBMs in clinical practise from the survey and focus groups is detailed below.

**Table 4:** Summary of focus group qualitative findings mapped to the TDF domains and barriers and enablers for AD BBM

Theme	Barrier/Enabler/Mixed	Frequency (total max *N* = 16)	Example quote(s)
TDF domain: KNOWLEDGE			
Understanding indications for test use	Mixed	13	‘I can think of specific case that you know I want to answer the question, neurodegenerative or not, that could be that could be one biomarker that could be useful.’ Focus group 1 (Enabler) ‘I think it will be really imperative to start with people who are clinically symptomatic, and the idea that they’ll be used initially, you know, for predicting conversion to dementia would be just too early in the evidence base for these biomarkers.’ Focus group 3 (Barrier)
Awareness of test and what the test measures	Mixed	10	‘I don’t know the state of the evidence on how a particular or what value of a particular result relates to the amount of tau pathology?.’ Focus group 3 (Barrier) ‘There’s very close correlation in 90% sort of levels between CSF and PET imaging and blood biomarkers now’. Focus group 3 (Enabler)
Awareness of test accuracy	Mixed	7	‘[…] the evidence to date is about this, you know, 75–80% accuracy.’ Focus group 2 (Enabler) ‘It’s not going to be always accurate in everybody.’ Focus group 3 (Barrier)
Equating pathology with a clinical diagnosis	Barrier	3	‘Where actually what we’re seeing is somebody with an MCI who is developing an Alzheimer’s pathology but doesn’t actually have the dementia? And I think that that is going to be a really a really important distinction to make’. Focus group 3 (Barrier)
TDF domain: SKILLS			
Communication of test information and result (limitations, legal ramifications, uncertainty)	Mixed	8	‘I think the challenge is communicating what is potentially quite complex in simple terms.’ Focus group 1(Barrier) ‘It’s just one more bit of information which will help us to find out what might be going on. Umm. . .so that’s one way that I could imagine the challenges of communication could sort of be eased.’ Focus group 3 (Enabler)
TDF domain: SOCIAL/PROFESSIONAL ROLE AND IDENTITY			
Role of the clinician to recommend a test as part of holistic assessment	Mixed	11	‘I think that we should be really cautious about using it before it’s been approved.’ Focus group 3 (Barrier) ‘[…]it’s just one of the many tests that we’re doing as part of the diagnostic workup. Umm. . .and so, you know in that sense, you don’t need to sort of torture the point too much of is this a diagnostic test for dementia.’ Focus group 1(Enabler)
Impact of blood biomarker upon professional identity	Mixed	4	‘You wouldn’t you know, you’ve got liver, you’ve got this type of breast cancer, or this type of liver disease without actually a diagnostic test which is taking you closer to understand that[. . .]Some parity with other medicine, really.’ Focus group 3 (Enabler) ‘So, we have two nurse prescribers in the team and then the background is mental health nursing. . .going to try to find another job because they feel threatened by. . .by of course, she doesn’t know much about them at the moment, but the approach has not been that medicalised.’ Focus group 3(Barrier)
Ethical use of finite resources	Barrier	3	‘I think that from thinking about kind of ethical standpoint, I guess there’s like a kind of financial NHS resources kind of ethical side.’ Focus group 3 (Barrier)
Healthcare professional role to provide reassurance/dispel myths	Barrier	2	‘I feel my job in memory clinic is to more to take away some of the myths that have always come.’ Focus group 1 (Barrier)
TDF domain: BELIEFS ABOUT CAPABILITIES			
Confidence of clinicians in using and interpreting the test	Mixed	7	‘I feel like it gives me like a sense of challenge.’ Focus group 4 (Barrier) ‘So I think that we’re probably fairly well equipped to have these kinds of discussions.’ Focus group 3 (Enabler)
Perceived capability of patients and clinicians to understand test-related information	Mixed	4	‘I actually think that the understanding of that, that biomarker-based diagnosis is relatively poor among lots of colleagues.’ Focus group 3 (Barrier) ‘[…]people have been able to put up with this complexity.’ Focus group 3 (Enabler)
TDF domain: OPTIMISM			
Clinicians are cautiously optimistic about this blood test	Enabler	9	‘So it is going to be a massive leap forward, but not a straightforward leap.’ Focus group 1 (Enabler)
TDF domain: BELIEFS ABOUT CONSEQUENCES			
Perceived benefit of the test to improve diagnostic certainty, accuracy or timing of diagnosis	Mixed	15	‘So we know that our accuracy of diagnosis is not great at the moment, so I think anything that comes in that could help us in that regard would be potentially valuable…’ Focus group 2 (Enabler) ‘It might nudge you a little bit, but it might be nudging you the wrong way.’ Focus group 2 (Barrier)
Concerns over the consequences of how different professionals and settings may use the blood test	Barrier	12	‘[…]we’ve got some brilliant neurologists, but they are very reductionist. And if they saw a positive test, their thinking would stop and it would just be, well that is definitely Alzheimer’s.’ Focus group 1 (Barrier)
If a blood test will improve access to current or future management or treatments	Mixed	10	‘I suppose there’s a big question around how useful is that going to be, given the lack of treatment options at the moment?’ Focus group 2 (Barrier) ‘I think it might make a difference in that circumstance in enabling access to treatment for people who probably in the vast, vast majority of cases, have a mixed pathology.’ Focus group 2 (Enabler)
False positives and negatives are a potential negative outcome of testing	Mixed	9	‘if you’re testing everyone, then you are going to deal with potentially a lot of err…overinclusion of a lot of potentially false positives.’ Focus group 1 (Barrier) ‘I mean every test is caveated isn’t it because it always, there will always be false negative false positives and that’s inherent in medicine, isn’t it.’ Focus group 2 (Enabler)
The biomarker result may conflict with other investigation information or the clinical impression	Barrier	4	‘I could imagine situations in which you do not think it is clinically likely that someone has dementia, but you unexpectedly get a positive result.’ Focus group 1 (Barrier)
The blood test is equally or more acceptable than other more invasive tests	Enabler	6	‘A blood-based biomarker is a lot easier than sending someone for a CSF biomarker potentially and a lumbar puncture.’ Focus group 1 (Enabler)
Concerns over possible consequences of a positive, intermediate or negative test result	Barrier	2	‘Is there a consequence on the insurance? Because will the insurance person know the difference between Alzheimer’s disease, MCI and Alzheimer’s dementia?.’ Focus group 4 (Barrier)
TDF domain: INTENTION			
Strong intention to use test	Enabler	4	‘100% likely I’m all in.’ Focus group 4 (Enabler)
TDF domain: GOALS			
It is a priority to have access to the test	Enabler	2	‘So, I would see it as a as a high priority to have access to it. That’s not the same as saying it’s a high priority to do it in everyone.’ Focus group 2 (Enabler)
TDF domain: MEMORY, ATTENTION AND DECISION MAKING			
Integration of test into current diagnostic algorithm	Mixed	11	‘I would be using it at the point where I would be using other biomarkers.’ Focus group 4 (Enabler) ‘I’m not also not certain on exactly where it sits in terms of a kind of algorithm of different investigations. Focus group 4 (Barrier)
TDF domain: ENVIRONMENTAL CONTEXT/RESOURCES			
Integrating test into existing systems	Mixed	9	‘It’s not something that we are currently set up for, but it would seem straightforward to integrate this into our diagnostic pathway.’ Focus group 1 (Enabler) ‘I think there’s difficulties with bloods in general in some places in my locality.’ Focus group 2 (Barrier)
Financial and resource constraints of test	Mixed	9	‘10s of pounds for a blood biomarker versus I know how many 100 pounds or thousands of pounds when you get to the logistics of organising a PET as well in a regional centre, it’s something that democratises.’ Focus group 2 (Enabler) ‘I think it be difficult to bring something like this in with how stretched things are. I think our budget has gone down absolute terms from the ICS in the last year.’ Focus group 2 (Barrier)
TDF domain: SOCIAL INFLUENCES			
Pressure from non-clinical stakeholders to receive testing	Enabler	7	‘I think there’s going to be a pressure from patients and their families who say, well, you’re telling me it’s probable Alzheimer’s, but can you be sure? Can you do more?’ Focus group 2 (Enabler)
Impact of perceived HCP authority on shared decision making	Enabler	3	‘Of course, your collaborative approach not a directive approach, but there are often situations when the question you know when the ball lands back in your court, asking what do you think Doctor?’ Focus group 3 (Enabler)
Enthusiasm of colleagues	Mixed	2	‘One thing that we haven’t really touched on is, you know, do we, are we, you know, is there the appetite locally?’ Focus group 1 (Mixed)
TDF domain: EMOTION			
Concern over the associated emotional impact of a test result for the patient, clinician or family	Mixed	11	‘There could be the risk of someone being disappointed or made, more anxious by a result that they can’t understand.’ Focus group 1 (Barrier) ‘I’m not excessively concerned that this is going to be something extra as a burden or emotional issue.’ Focus group 1 (Enabler)
TDF domain: BEHAVIOURAL REGULATION			
Impact of test on diagnostic procedure	Mixed	2	‘I think understanding these tests and how it might feedback on diagnostic practise will be really interesting.’ Focus group 3 (Mixed)

#### Behavioural regulation

Behavioural regulation, reflecting self-reflection and monitoring processes, was identified as a key enabler, with 95.3% of respondents agreeing that comparing use of AD BBMs with local colleagues, and 88.4% agreeing that developing local policy for test use, would enable implementation of the blood biomarker test.

In focus groups, participants were interested to understand how the test would impact diagnosis: *‘[…] how it might feedback on diagnostic practice will be really interesting’* (Participant 12, Consultant).

#### Social professional role and identity

‘Social/Professional Role and Identity’, reflects a HCP’s perception of their professional ethical standards, and role boundaries. This domain contained both enablers and barriers. In the survey responses, 82.0% of respondents felt professionally responsible for adopting new diagnostic technologies, while 70.9% agreed that clinicians should use AD BBMs to improve their diagnostic accuracy. However, 48.3% agreed that delays between approval and guidance for use of AD BBMs could hinder their use, reflecting uncertainty about professional accountability and role boundaries when clear implementation guidance is lacking.

This was echoed in the focus groups as a barrier to use: *‘I think that we should be really cautious about using it before it’s been approved’* (Participant 10, Consultant). Open-text responses and focus groups identified a barrier being a need to ensure the test did not supersede the clinician’s role in completing a robust clinical assessment *‘Clinical assessment will always be gold standard’* (open-text survey response). Clinicians’ concerns about ethically allocating finite limited resources for AD BBM testing were a barrier in this domain, highlighting the sense of professional duty to ensure fair and appropriate use.

#### Emotion

The ‘Emotion’ domain, reflecting affective responses influencing behaviour, was a mixed barrier/enabler to AD BBM use. In the survey responses, it was generally identified as an enabler to test use, with positive feelings toward AD BBMs reported by 76.7% of respondents, and few reporting frustration (3.5%) or fear of the test replacing their expertise (4.1%)*.*

However, in the focus groups, the emotional burden of testing was identified as both a barrier and enabler to use, with a barrier identified as: *‘There could be the risk of someone being disappointed or made more anxious by a result that they can’t understand’* (Participant 2, Specialty Registrar).

#### Memory, attention and decision processes

This domain, concerning cognitive focus and decision-making, emerged as an overall enabler, with only 20.9% of survey respondents indicating that AD BBMs would significantly increase decision-making effort, suggesting most did not anticipate finding the test burdensome.

Integrating the blood test into the current diagnostic algorithm was a mixed barrier/enabler for focus group participants: *‘I’m also not certain on exactly where it sits in terms of a kind of algorithm of different investigations’* (Participant 13, Consultant)*.*

#### Optimism

‘HCPs’ positive expectations about outcomes, was overall an enabler to AD BBM implementation, with 84.3% of survey respondents optimistic that AD BBMs would improve Alzheimer’s dementia diagnosis, and only 18.0% doubting treatment impact. However, optimism about the test’s accuracy was moderate, with only 52.9% believing BBMs are highly accurate for detecting AD pathology.

In the focus groups there was cautious optimism about the blood test: *‘So it is going to be a massive leap forward, but not a straightforward leap’* (Participant 5, Specialty and Specialist doctor)*.*

#### Social influences

The impact of social norms and peer expectations on behaviour, was predominantly an enabler to test use, with most survey respondents indicating AD BBMs would be positively received by both patients (71.5%) and their families (75.6%). Additionally, 70.3% said access to AD BBMs would boost their own diagnostic confidence.

In the focus groups, participants perceived pressure from non-clinical stakeholders as an enabler to testing: *‘I think there’s going to be a pressure from patients and their families who say, well, you’re telling me it’s probable Alzheimer’s, but can you be sure? Can you do more?’* (Participant 8, Consultant)*.*

#### Reinforcement

‘Reinforcement,’ which concerns rewards or incentives that strengthen motivation to perform a behaviour, served predominantly as an enabler to implementation. Survey respondents indicated the potential for AD BBM use to be intrinsically rewarding, with 70.3% agreeing that the test would enhance personal confidence in their diagnostic skill. Evidence of negative reinforcement was very limited, with only 1.2% reporting they would avoid using the test due to concerns about distressing patients, suggesting a minimal disincentive to test use.

No barriers or enablers related to the Reinforcement domain were identified in the focus groups.

#### Goals

‘Goals,’ which relate to HCPs’ prioritisation and commitment to specific objectives, was a barrier to test implementation, as only 34.3% of survey respondents agreed with the statement: ‘My personal target is to reduce the number of patients diagnosed with MCI.’, suggesting that refining diagnostic subtyping is not currently a high personal or service priority for most HCPs. Additionally, only 23.3% agreed that their service had targets focused on increasing the proportion of patients with a pathology-based dementia diagnosis.

In the focus groups a participant identified being able to perform the AD BBM test when appropriate, as a priority: *‘So, I would see it as a as a high priority to have access to it. That’s not the same as saying it’s a high priority to do it in everyone’* (Participant 7, Consultant).

#### Knowledge

‘Knowledge’ was identified as a barrier, with only 47.1% of survey respondents being familiar with the AD BBM evidence base, 55.2% understanding what these biomarkers measure, and 26.7% aware of relevant appropriate use criteria. Open-text survey responses highlighted ‘*Lack of awareness’* as a barrier and focus groups emphasised a lack of awareness of what the test measures and test accuracy: *‘It’s not going to be always accurate in everybody.’* (Participant 12, Consultant). Additionally, equating pathology with diagnosis was identified as a barrier in both open-text survey responses and focus groups: *‘The biggest challenge in my view is the distinction between Alzheimer’s pathology and Alzheimer’s disease.’* (Open-text survey response).

#### Skills

The ‘Skills’ domain, reflecting an ability acquired through practise, was a mixed barrier/enabler to test implementation, with 72.1% of survey respondents indicating they were confident in communicating the result to a colleague and 71.5% confident in communicating this to a patient or family member. Conversely, in open-text responses, and focus groups this was identified as a barrier to testing: *‘The challenge will be explaining the limitations of the tests’* (Open-text survey response) and *‘I think the challenge is communicating what is potentially quite complex in simple terms’* (Participant 5, Trainee).

#### Environmental context and resources

Organisational and resource-related influences were largely barriers to AD BBM implementation, with only 45.9% of survey respondents indicating access to a phlebotomy service, 45.9% to blood transportation and 48.8% to a lab for blood sample analysis. Only 24.4% reported access to all three resources. Open-text survey responses and focus groups identified integration into existing systems and pathways as a barrier within the theme ‘Systems and Pathways’: *‘The blood biomarker test may delay the diagnostic process.’* (open-text survey response). ‘Lack of resources’ was another barrier: *‘We just lack the infrastructure.’* (open-text survey response). However, focus groups also identified the test’s cost-effectiveness as an enabler: *‘It’s something that democratises.’* (Participant 8, Consultant).

#### Beliefs about capabilities

Confidence in one’s own ability to perform a behaviour, was overall a barrier to implementation. Confidence in clinical application was variable, with 53.5% confident in the use of AD BBMs and 57.0% confident in the interpretation of results. However, only 16.9% believed allied health professionals could effectively use AD BBMs in diagnosis*.* This was reflected in the focus groups with using and interpreting AD BBMs identified as a *‘sense of challenge’* (Participant 15, Trainee) and concern over perceived capability of colleagues to understand test-related information: *‘the understanding of that, that biomarker-based diagnosis is relatively poor amongst lots of colleagues’* (Participant 10, Consultant)*.*

#### Beliefs about consequences

Perceptions of the likely outcomes of a behaviour, revealed mixed enablers and barriers to AD BBM implementation. Survey respondents saw AD BBMs as offering diagnostic guidance (87.8%), reassuring patients (86.6%), and enabling earlier diagnosis (82.6%), but reported concerns about indeterminate results (82.6%), discrepancies with clinical presentation (80.2%), and inconsistencies with other diagnostic tests (72.1%) (Table 3). Open-text survey responses echoed these concerns: *‘The test may not align with clinical findings.’* Focus groups also identified diagnostic benefits, such as improving diagnostic certainty, but highlighted mixed views on treatment access*: ‘How useful is that going to be, given the lack of treatment options?’* (Participant 6, Consultant). Others saw test utility and treatment availability as distinct issues, or as enabling access to treatment for patients with mixed pathology: *‘It enables access to treatment for people who, in the vast majority of cases, have mixed pathology.’* (Participant 8, Consultant). In the focus groups, a blood test was perceived as more acceptable than more invasive forms of testing. Concerns about AD BBM test result implications (e.g. insurance coverage, varied professional use) were barriers: *‘Is there a consequence on the insurance?’* (Participant 14, Consultant).

#### Linear regression analyses

Survey respondents had strong ‘Intentions’ to use the test, with 93.0% intending to use the test in clinical practise if it were approved and available for use. To examine which TDF domains were associated with intention, as a proxy for the behaviour (i.e. implementing AD BBMs), a multiple linear regression model was fitted. The regression model was significant (*R*^2^ = 0.43). Model assumptions were met (Breusch-Pagan: *P* = .13; Durbin-Watson: DW = 1.93, *P* = .64; VIF = 2.33; Shapiro–Wilk: *P* = .08).

‘Memory, attention and decision processes’ (B = 0.44, 95% CI 0.20 to 0.68), ‘Beliefs about Consequences’ (B = 0.45, 95% CI 0.11 to 0.78) and ‘Social Influences’ (B = 0.24, 95% CI 0.04 to 0.44) were positively associated with ‘Intentions.’ ‘Optimism’ (B = -0.31, 95% CI -0.58 to −0.04) and ‘Emotion’ (B = -0.33, 95% CI -0.60 to −0.06) were negatively associated. Other TDF domains were not significantly associated with intention. A full summary of regression coefficients, standard errors and *P*-values is provided in Appendix S9. Associations remained significant after adjustment for consultant role and geographic region (Appendix S10).

### Interventions

#### Intervention functions

Survey respondents strongly supported most intervention functions for AD BBM implementation, with highest agreement for ‘Guidelines’ (Training/Education, 98.1%), ‘Changes Needed Within the Service Environment’ (Environmental Restructuring, 95.5%), ‘Further Education’ (Education, 94.5%) and ‘Training’ (Training, 94.5%), but lower agreement for ‘Incentives’ (Incentivisation, 52.3%) (Appendix S11). Open-text responses (*n* = 23) mapped to seven BCW intervention functions, notably ‘Environmental Restructuring’ (e.g. logistical concerns) and ‘Education’ (e.g. guidance for clinicians and patients) (Appendix S12). A summary of focus group qualitative findings mapped to the intervention functions in the BCW is presented in Table 5.

**Table 5:** Summary of focus group qualitative findings mapped to the intervention functions in the BCW

Theme	Frequency (total max *N* = 16)	Example quote(s)
**Intervention Function(s): Education** (Increasing knowledge or understanding)		
Information on biomarker properties (purpose, accuracy, limitations) and test delivery e.g. logistics	12	‘What is it measuring? Just like a reminder of the basics?’ ‘If you are going to take the blood, what tube is it?’
Guidelines on appropriate use criteria	6	‘[…]the main sort of piece of education that would be helpful is when to use it.’
Evidence base for test	4	‘[…] so with this particular biomarker, the original research papers having the links to them so people could do a deeper dive into the data would be useful.’
Appropriateness of language, content and relevance to intended audience	2	‘So we are talking about someone who did not have the framework of a, you know, medical school or who did not have the framework of medical training. So it definitely has to be has to be tailor made for that.’
**Intervention Function(s): Education** (Increasing knowledge or understanding) **& Training** (Imparting skills)		
Test result interpretation and application	6	‘[…] people will want to know about them as well as the interpretation of the test.’
Communication of test result to a patient	5	‘I mean potentially something about how to interpret and explain the result to the patients, people might find useful.’
Communicating uncertainty and managing patient expectations	2	‘So I think it would be something about highlighting maybe the downsides that might be there when you talk to that individual and how much reliability we can maybe ascribe to the result.’
**Intervention Function(s): Enablement** (Increasing means/reducing barriers to increase capability or opportunity)		
User-friendly design	8	‘But the newer stuff incorporates videos, quizzes, different, different ways of navigating through content.’
Dynamically updated diagnostic algorithm	5	‘I suppose maybe people might want to understand the difference between when you would use a blood biomarker test to CSF as opposed to PET and understand where it fits in the decision pathway.’
Accurate and up-to-date content	4	‘Good is when they’re up to date.’
**Intervention Function(s): Environmental restructuring** (Changing the physical or social context)		
Multimodal delivery	2	‘I think people would be happy to attend seminars, you know, face to face or online, updated teaching and an e-module certainly would be very reasonable.’
Text and visual aids to support discussions	6	‘They are going to want to have a simple plain English summary which they can deliver to the patient.’
Accessibility of tool	6	‘Make it free.’
Access to training through existing institutions	2	‘[…] being able to be incorporated into existing NHS structures is going to be the most important thing.’ ‘Not requiring lots of passwords and new like accounts.’
Local teaching and audit/multidisciplinary team reviews	1	‘We need to reflect on how this is being used, have local learning, have local clinical reviews on how its working out.’
**Intervention Function(s): Incentivisation** (Creating an expectation of reward)		
Gain CPD points through e-learning	4	‘[…] so that people could use it for CPD purposes.’
**Intervention Function(s): Modelling** (Providing an example for people to aspire to or imitate)		
Showcase best practises using case scenarios for incorporating the test results clinical assessments	3	‘I like the ones about, that would take you through the clinical process and you know you have this case scenario which you recognise from a clinical practise and this is how you use them and this is how it helps you to diagnose this.’
Demonstrate effective pre-test counselling and result communication techniques	4	‘I think it could be quite useful to have examples with how a clinician might explain the different types of results to patients in the clinical context in the vignette.’
**Intervention Function(s): Persuasion** (Using communication to induce positive feelings or negative feelings or stimulate action)		
Patient/carer testimonials	4	‘[…] I think it would be good to have the patient testimonials to get the patient perspective.’
Highlight success stories of clinicians effectively using ad biomarkers to improve patient care	1	‘It could be, so it could be doctors talking about how they use it in their day-to-day practise.’
Accredited/validated by credible source	4	‘I’d really want to be doing some kind of robust approved CPD around this’.
Balanced presentation of information (strengths and weaknesses of testing)	1	‘I think that what this all makes me think is that they need to be really downplayed and almost the training needs to be very cautiously optimistic maybe.’
**Intervention Function(s): Restriction** (Using rules to reduce the opportunity to engage in the target behaviour)		
Licence to test	4	‘[…] if it was something for me that gave me the green light that I was safe and competent to use it, then yeah, I’d be interested in doing it.’

### Percentage agreement service comparison

Percentage agreements with belief statements or intervention strategies were not statistically significantly different between psychiatrists who worked in memory services or another service type, after Bonferroni correction (Appendix S11 and S13). Similarly, no significant difference were observed in belief statement agreement between academic and non-academic centres (Appendix S14).

## Discussion

This study built on our systematic review [18], using the TDF to identify psychosocial and contextual barriers and enablers to AD BBM use among UK old age psychiatrists. Findings aligned with the review, emphasising ‘Knowledge’ and ‘Beliefs about Consequences’ as key domains, while revealing new barriers and enablers and confirming strong agreement with proposed intervention functions.

‘Behavioural Regulation’ emerged as an enabler, e.g. using audit and feedback to standardise practise, a well-established strategy in dementia care [27], and a routine part of psychiatrists’ professional appraisal [28]. High agreement within the domains of ‘Intentions’ (e.g. intention to use the test)*,* and ‘Optimism’ (e.g. belief in improving AD diagnosis); represented generally positive views toward AD BBMs, despite limited exposure [4], possibly drawing on experience with other biomarkers [29]. However, challenges such as interpreting intermediate results [30] limit direct comparisons. Ratings of belief statements did not vary by work setting, likely reflecting similar resource constraints across community services [31].

Another barrier was in the TDF domain ‘Goals,’ with low percentage agreement regarding personal or service targets for dementia subtyping. This corresponds with under-recording of subtyped diagnoses [32] and could represent limited knowledge of the test’s discriminatory value, or reduced prioritisation of precision diagnosis. Low agreement with incentivisation via national targets supports this interpretation. Interventions should emphasise benefits of precise diagnoses, including enhanced access to targeted treatments and clearer prognostic information [33].

Another key barrier was seen within the TDF domain ‘Knowledge,’ including limited awareness of appropriate use criteria, likely due to fragmented guidance [34, 35]. Free text response and focus groups highlighted challenges with test interpretation, consistent with previous literature [36], suggesting that enhancing confidence in result interpretation could promote AD BBM uptake [37]. High agreement with interventions to support further education, and training, is consistent with published recommendations for implementation [38].

Previous surveys have explored the barriers to AD biomarker use, [16, 39, 40] but mostly focused on CSF and amyloid-PET, across HCPs in primary and secondary care and without using a structured framework like the TDF. Despite this, their findings also highlight concerns about psychological impact for patients and lack of disease-modifying treatments (‘Beliefs about consequences’ domain) [39]. As one respondent in our survey noted: *‘What is the benefit at this time of knowing you are developing AD when there is really jack all we can do about it?’*. However, psychiatrists also see the value in biomarker tests for providing objective evidence of pathology, thereby supporting AD diagnoses [5], underscoring the complexity of decision-making around BBM use.

Focus groups further emphasised barriers in the TDF domain ‘Environmental Context and Resources’, including an absence of pathways and systems to deploy BBMs in community services*.* Clinicians perceive challenges in integrating the test into daily practise [41]. Similar concerns have been reported internationally [42, 43] and reflect wider-system readiness challenges [43] and the absence of a streamlined AD diagnostic pathway [16].

### Strengths and limitations of the study

This is the first mixed-method study to frame the use of AD BBMs around the TDF and BCW and incorporate UK psychiatrists’ perspectives, offering a theoretically-informed understanding of behavioural and contextual determinants. Many implementation initiatives can lack theoretical grounding, and lead to poor translation of new innovations, evidence or technologies into clinical practise. Both the quantitative and qualitative work proactively identified barriers and enablers to support effective implementation and AD BBMs and identified targeted implementation strategies, informed by prominent TDF domains and stakeholder input through ratings of proposed interventions.

Limitations include a modest sample size (172), comparable to similar surveys [44], but with potential selection bias and limitations in generalising the study findings. More motivated clinicians were likely to complete the survey. The cross-sectional design limits causal inference. The low response rate (3.3%) raises the possibility that non-respondents may hold different views than respondents and that the topic was not of broader interest. A strength of the focus groups was the ability to explore the survey findings in further depth, attempting to capture the complexities that a cross-sectional survey cannot fully address. However, a small focus group sample (4 groups, *N* = 16) may limit transferability.

The overrepresentation of academic centres, consultants in England and respondents whose clinical practise involved a lower proportion of patients with AD than average may reduce the generalisability and representativeness of the findings to the target survey population of old age psychiatrists and, more broadly, NHS clinicians who may use AD BBMs. Survey length may have contributed to the relative high dropout rate, though we deemed this necessary to address all the TDF domains.

Intention was used as a proximal outcome measure to assess the potential for future uptake. However, the intention–behaviour gap highlights that intention does not always translate into actual behaviour [45]. Contextual constraints, competing priorities, and systemic barriers can prevent individuals from acting on their intentions, even when motivation is high. Future work should include follow-up data on actual clinical behaviours once implementation begins.

### Implications for clinical practise and policy

These study findings can inform the development of intervention strategies to facilitate the integration of BBM testing into practise by addressing clinician related factors identified. Despite identified barriers, respondents had a high intention to use the AD BBMs if made available. In the regression analyses, ‘Memory, Attention and Decision Processes’, ‘Beliefs about Consequences’ and ‘Social Influences’ were positively associated with intention. This suggests that clinicians’ cognitive processing, perceived benefits of testing and perceived professional attitudes or peer norms may shape their motivation to adopt AD BBMs.

In contrast, ‘Emotion’ and ‘Optimism’ were negatively associated with intention, reflecting a more nuanced perspective that aligns with the qualitative findings and may reflect increased confidence in clinical diagnosis making among some clinicians. While overall belief ratings were positive, concerns regarding the emotional burden of testing were identified. Similarly, optimism was expressed in a cautious or conditional manner, which suggests respondents may weigh anticipated benefits against other factors such as system readiness or available treatment options.

Other domains, such as ‘Knowledge’ and ‘Environmental Context and Resources’, while theoretically relevant, did not show significant associations in this model. This may indicate that intention is more strongly influenced by internal cognitive and reflective factors than by external resources or information alone.

Interventions aiming to increase AD BBM use may benefit from supporting decision-making processes (e.g. through clinical decision support tools or training simulations), clear communication of the potential benefits of testing and reporting peer attitudes to testing. Further, addressing potential emotional outcomes of testing and framing language in a balanced manner, may support implementation.

When asked to rate intervention strategies for supporting AD BBM use, respondents gave most strategies their strong support, indicating broad enthusiasm but limiting the ability to identify a specific, targeted intervention.

The highest agreement was for the development of national appropriate use guidelines. These could provide updated guidance on those previously published [38], in light of advancements in understanding of assay performance and clinical utility of AD BBMs and led via collaboration between leading UK health and dementia organisations.

Wider policy is needed to address environmental barriers, with respondents supporting initiatives to make changes to the service environment. Services will need to be resourced and organised to ensure there are systems and pathways in place to implement AD BBMs. Clinical pathways for AD BBM use should build on global recommendations [46], supported by national policy and NHS England guidance. NHS England is already proactively planning system readiness for any future approval of disease-modifying treatments, which includes integrating biomarker testing into healthcare system readiness [47].

## Conclusions

This study identified barriers and enablers to AD BBM implementation across TDF domains, highlighting diagnostic benefits and resource challenges. Generalisability of study findings must be interpreted cautiously in light of the low response rate. Next steps involve formal triangulation of study findings and co-design using the BCW to map barriers/enablers and intervention strategies, to inform the design of an intervention to support their implementation.

## Supplementary Material

Supplementary_materials_afag117

## Data Availability

The data that support the findings of this study are available from the corresponding author upon reasonable request, within privacy/ethical restrictions.
