# Correction to: Factors influencing implementation of an Alzheimer’s disease blood test among UK old age psychiatrists: mixed-methods study using the theoretical domains framework

**DOI:** 10.1093/ageing/afag146

**Published:** 2026-05-14

This is a correction to: Jemma Hazan, Mitchell Mealing, Fabiana Lorencatto, Penny Rapaport, Ashvini Keshavan, Jonathan M Schott, Joanne Rodda, Robert Howard, Factors influencing implementation of an Alzheimer’s disease blood test among UK old age psychiatrists: mixed-methods study using the theoretical domains framework, *Age and Ageing*, Volume 55, Issue 5, May 2026, afag117, https://doi.org/10.1093/ageing/afag117

The first author's names were originally transposed in error, and have been corrected in the paper to read: “Jemma Hazan”.

