# Supplementary material for: Factors influencing implementation of an Alzheimer’s disease blood test among UK old age psychiatrists: mixed-methods study using the theoretical domains framework
Source: Age Ageing. 2026 May 6;55(5):afag117. doi: 10.1093/ageing/afag117 (PMC13147441; doi:10.1093/ageing/afag117)
Supplement: Supplementary_materials_afag117 [file supplementary_materials_afag117.zip › Supplementary_materials_afag117_File002.docx]

**Factors Influencing Implementation of an Alzheimer’s Disease Blood Test among UK Old Age Psychiatrists: a Theoretical Domains Framework-based Mixed-methods Study**

**Appendices**

**Contents**

**Appendix 1 – Survey Questions pdf – uploaded separately**

**p.2 Appendix 2 - Extraction of quantitative data**

**Appendix 3 – Focus Group Topic Guide-uploaded separately**

**p.3 Appendix 4 – Step-wise process used for thematic analysis.**

**p.5 Appendix 5 – Additional demographic survey data**

**p.6 Appendix 6- Key demographic details of focus group participants**

**p.7 Appendix 7- Current practices in AD diagnosis amongst survey respondents**

**p.9 Appendix 8- Summary of Open-text Survey Response Qualitative Findings Mapped to the TDF domains and Barriers and Enablers for AD BBM Testing**

**p.12 Appendix 9- Linear regression analysis examining the association between TDF domains and intention**

**p.13 Appendix 10-** **Sensitivity analysis: Multiple linear regression predicting intention, additionally adjusted for consultant status and geographic region**

**p.14 Appendix 11- Mean scores and percentage agreement with belief statements representing intervention functions for AD BBM use by service (memory service vs other service)**

**p.16 Appendix 12- Summary of Survey Open-text responses for strategies to improve AD BBM Testing mapped to the intervention functions in the Behaviour Change Wheel.**

**p.18 Appendix 13- Mean scores and percentage agreement with belief statements representing barriers and enablers to AD BBM use by service (memory service vs other service)**

**p.24 Appendix 14-Mean scores and percentage agreement with belief statements representing barriers and enablers to AD BBM use by service (academic vs non-academic centre)**

| **Section** | **Numerical values assigned to Likert:** | |
| --- | --- | --- |
|  | **Strongly Agree** | **Strongly Disagree** |
| 3 | 5 | 1 |
| 4 | 3 | -3 |
| 5 | 5 | 1 |

**Appendix 2. Extraction of quantitative data from statements in Sections 3, 4, and 5.**

| **Step** | **Activity** |
| --- | --- |
| 1 | Researchers repeatedly read transcripts to familiarise themselves with the data. |
| 2 | A codebook, based on the 14 TDF domains, was developed by JH and reviewed by an experienced Behavioural Scientist (FL) to guide deductive coding, including domain definitions and examples. |
| 3 | Deductive framework analysis: Two trained researchers (JH & MM) independently coded participant responses to the most relevant TDF domain(s), analysing transcripts sequentially. Responses could be coded to multiple domains where applicable. |
| 4 | Inductive thematic analysis: JH and MM grouped responses by TDF domain, generating theme labels to summarise their role in influencing AD BBM use. FL reviewed labels for accuracy and domain fit, resolving discrepancies through discussion. Themes were classified as barriers, enablers, or mixed influences. |
| 5 | *BCW Interventions*  Two trained researchers (JH & MM) independently coded participant responses to the most relevant BCW intervention function(s), analysing transcripts sequentially.  Responses were grouped by BCW intervention functions and inductively analysed to form themes. |

**Appendix 4. Step-wise process used for thematic analysis.**

| **Demographics** | | n | % of total |
| --- | --- | --- | --- |
| **Years practising Old Age Psychiatry** | 1 - 5 | 46 | 26.7 |
|  | 6 - 10 | 26 | 15.1 |
|  | 11 - 15 | 22 | 12.8 |
|  | 16 - 20 | 42 | 24.4 |
|  | 20+ | 36 | 20.9 |
| **Role** | Consultant | 109 | 63.4 |
|  | Specialty Trainee | 34 | 19.8 |
|  | Core Trainee | 4 | 23.3 |
|  | Specialty and Associate Specialist (SAS) Doctor | 25 | 14.5 |
| **Trained in other specialty** | Yes | 58 | 33.7 |
|  | No | 114 | 66.3 |
| **Specialty Trained In** | General Medicine | 20 | 34.5 |
|  | Neurology | 13 | 22.4 |
|  | General Practice | 6 | 10.3 |
|  | Emergency Medicine | 1 | 0.58 |
|  | ENT Surgery | 1 | 0.58 |
|  | Geriatric Medicine | 2 | 1.16 |
|  | Neurosurgery | 1 | 0.58 |
|  | Urology | 1 | 0.58 |
|  | Other | 13 | 7.56 |
| **Region** | England | 144 | 83.7 |
|  | Northern Ireland | 3 | 1.7 |
|  | Scotland | 18 | 10.5 |
|  | Wales | 7 | 4.1 |
| **(Participants in England) Sub-Region Practised In** | East Midlands | 12 | 7.0 |
|  | East of England | 11 | 6.4 |
|  | London | 22 | 12.8 |
|  | North East | 18 | 10.5 |
|  | North West | 22 | 12.8 |
|  | South East | 17 | 9.9 |
|  | South West | 17 | 9.9 |
|  | West Midlands | 11 | 6.4 |
|  | Yorkshire and The Humber | 14 | 8.1 |
| **Works in Academic Centre** | Yes | 86 | 50.0 |
|  | No | 86 | 50.0 |
| **Service** | Inpatient setting | 73 | 42.7 |
|  | Community Mental Health Team (CMHT) | 114 | 66.3 |
|  | Memory Service | 107 | 62.2 |
|  | Liaison Service | 29 | 16.9 |
|  | Other | 13 | 7.6 |

**Appendix 5. Additional demographic survey data.**

| **Demographics** | | n | % of total |
| --- | --- | --- | --- |
| **Role** | Consultant | 11 | 69% |
|  | All other grades | 5 | 31% |
| **Region** | England | 14 | 88% |
|  | Northern Ireland | 0 | 0% |
|  | Scotland | 1 | 6% |
|  | Wales | 1 | 6% |
| **Years practising Old Age Psychiatry** | 1 - 5 | 9 | 56% |
|  | 6- 10 | 0 | 0% |
|  | 11-15 | 2 | 13% |
|  | 16-20 | 2 | 13% |
|  | 20+ | 3 | 19% |
| **Years working in current service** | 1 - 5 | 10 | 63% |
|  | 6- 10 | 2 | 13% |
|  | 11-15 | 2 | 13% |
|  | 16-20 | 0 | 0% |
|  | 20+ | 2 | 13% |
| **Service** | Memory service | 10 | 63% |
|  | All other | 6 | 38% |

**Appendix 6.** **Key demographic details of focus group participants.**

| **Current Practices** | | n | % of total |
| --- | --- | --- | --- |
| **Frequency HCP makes an AD diagnosis** | More than five a week | 87 | 50.6 |
|  | Between one and five a week | 20 | 11.6 |
|  | Less than once a week | 53 | 30.8 |
|  | Once a month or less | 12 | 7.0 |
| **Proportion of HCP’s practice involving patients with Alzheimer’s disease** | Less than 25% | 79 | 45.9 |
|  | About 25-50% | 28 | 16.3 |
|  | More than 50% | 65 | 37.8 |
| **Investigations HCPs routinely use when diagnosing Alzheimer's disease** | Clinical evaluation (history, examination and cognitive assessment e.g. MOCA/MMSE/ACE-III) | 172 | 100.0 |
|  | Referral to Neuropsychologist for neuropsychological assessment | 83 | 48.3 |
|  | Neuroimaging: MRI | 119 | 69.2 |
|  | Neuroimaging: CT | 142 | 82.6 |
|  | Neuroimaging: Amyloid PET | 13 | 7.6 |
|  | Neuroimaging: DAT scan | 67 | 39.0 |
|  | Cerebrospinal fluid (CSF) | 0 | 0.0 |
|  | Other | 18 | 10.5 |
| **Investigations HCPs routinely use when diagnosing Alzheimer's disease (Other)** | FDG PET | 5 | 27.8 |
|  | Genetic Testing | 1 | 5.6 |
|  | SPECT | 2 | 11.1 |
|  | OT completing AMPS | 1 | 5.6 |
|  | General Bloods | 1 | 5.6 |
| **Proportion of patients for whom HCP orders AD biomarker investigations** | 0% | 116 | 67.4 |
|  | <10% | 55 | 32.0 |
|  | 10-25% | 1 | 0.6 |
| **Recorded reasons to order specialist amyloid biomarker investigations** | To increase certainty of a diagnosis of Alzheimer’s disease | 77 | 47.2 |
|  | When the patient has a young onset (for example <65 years) | 129 | 79.1 |
|  | When the clinical presentation is atypical | 128 | 78.5 |
|  | To investigate the underlying aetiology of Mild Cognitive Impairment or dementia | 65 | 39.9 |
|  | Other: please specify | 21 | 12.9 |
| **Percentage of patients HCP refers to Neurology to establish a diagnosis** | 0% | 24 | 14.0 |
|  | <5% | 117 | 68.0 |
|  | 5-10% | 24 | 14.0 |
|  | >10% | 7 | 4.1 |
| **Percentage of HCPs who have ordered an Alzheimer’s disease CSF biomarker or amyloid-PET in clinical practice.** | Yes | 44 | 25.6 |
|  | No | 126 | 73.3 |
|  | Unsure | 2 | 1.2 |

**Appendix 7. Current practices in AD diagnosis amongst survey respondents.**

| **Subtheme** | **# of statements** | | | | **TDF Domain(s)** | **Representative Statements^a^** |
| --- | --- | --- | --- | --- | --- | --- |
|  | **Barriers** | **Enablers** | | **Mixed** |  |  |
| **THEME: Importance placed on testing** | | | | | |  |
| Current organisational culture | 1 | 0 | 1 | | Environmental Context and Resources,  Social Influences | ‘Reluctance by commissioners in adopting blood biomarkers for investigations’ ^B^  ‘Culture of memory clinics’ ^M^ |
| Biomarker weighting along the diagnostic continuum | 2 | 2 | 0 | | Beliefs about Consequences,  Social/Professional Role and Identity | ‘The best value for the biomarker would be in pre-senile dementias’ ^E^  ‘Clinical assessment will always be gold standard’ ^B^ |
| Perceptions of treatment | 1 | 1 | 0 | | Beliefs about Consequences | ‘In my view this is all aimed at establishing a diagnosis as early as possible, in particular as disease modifying Rxs^1^ become available over the coming years’ ^E^  ‘What is the benefit at this time of knowing you are developing AD when there is really jack all we can do about it?’ ^B^ |
| Limitations to testing | 5 | 0 | 0 | | Beliefs about Consequences | It must be stressed that incorporation of this test in routine practice will not necessarily increase treatment efficacy’ ^B^  ‘Diagnostic accuracy likely to wane with older patients’ ^B^ |
| **THEME: Testing can have both beneficial and adverse outcomes** | | | | | |  |
| Beneficial outcomes of testing | 0 | 5 | 0 | | Optimism,  Social Influences,  Beliefs about Consequences | ‘The develop of their utility in diagnosis however is a very welcome step forward.’ ^E^  ‘Patients are already looking forward to it’ ^E^  ‘Opportunity to distinguish between Alzheimer’s and MCI^2^ or other dementias.’ ^E^ |
| Drawbacks of testing | 9 | 0 | 0 | | Beliefs about consequences | ‘It’s also very important to recognise that the vast majority of diagnoses are straightforward and based on symptomatology - there is no role for a test with 80% spec/sens^3^ in the vast majority of cases…’ ^B^  ‘Challenge of having a positive test when memory symptoms are not severe enough to diagnose a dementia’ ^B^  ‘Legal implications and disclosure advice for a positive blood results’ ^B^ |
| **THEME: Systems and pathways** | | | | | |  |
| Referrals | 1 | 1 | 0 | | Environmental Context and Resources | ‘I would hope it can rule out Alzheimer’s in most cases and that could help reduce the influx of worried well patients’ ^E^  ‘Huge surge in number of referrals to memory clinics, increasing the waiting time for those who deserve.’ ^B^ |
| Integration of test into clinical workstream | 2 | 0 | 0 | | Environmental Context and Resources | ‘There is a pressure to make a dementia diagnosis within 6 weeks of referral in our service and waiting for the blood bio marker test may delay this process although I feel it would be helpful when thinking about making an accurate diagnosis’ ^B^ |
| Lack of resources | 8 | 0 | 0 | | Environmental Context and Resources | ‘service provision and staffing levels in NHS^4^’ ^B^  ‘we just lack the infrastructure to make it feasible - we struggle to get clinic rooms and we have no one who can take bloods’ ^B^  ‘sufficient funds from commissioners’ ^B^ |
| Acceptability of testing | 1 | 2 | 0 | | Memory, Attention and Decision Making Processes | ‘Patients who have needle phobia’ ^E^  ‘… And the myth that LP^5^ is simple and painless is nonsense- I have done many LPs and had one myself and they hurt. FDG-PET^6^ scans are reported badly and always come back with vague non-specific findings and have a massive carbon footprint. So yes a blood test would be great...’ ^B^ |
| Accessibility of testing | 0 | 1 | 0 | | Beliefs about consequences, Environmental Resources | ‘Overall I feel quite positive about the test as currently I would like to be able to order LPs on patients presenting with atypical symptoms, but I do not have access to this service. If the BBMs were equivalent to an LP, then they would be very helpful for my Young Onset Dementia Service.’ ^E^ |
| **THEME: Training needs** | | | | | |  |
| Understanding appropriate use criteria for testing | 2 | 0 | 0 | | Knowledge,  Beliefs about Consequences | ‘The biggest challenge in my view is the distinct between Alzheimer’s pathology and Alzheimer’s disease. The risk and service pressure of treating those who are preclinical.’ ^B^ |
| Test Properties | 4 | 0 | 0 | | Knowledge | ‘I do not have enough information at the moment to understand how useful the blood biomarkers would be, in terms of sensitivity and specificity’ ^B^  ‘Lack of validation in real world settings – particularly complex patients with multiple comorbidities’ ^B^ |
| Communication | 3 | 0 | 0 | | Skills | ‘The challenge will be explaining the limitations of the tests and also how they do not account for the presence of co-pathology’ ^B^ |
| Test interpretation | 5 | 3 | 0 | | Knowledge,  Skills,  Beliefs about Capabilities | ‘I think there would have to be a robust e-learning package prior to their use as I feel there will be high variation in the confidence colleagues have in using these tests’ ^E^  ‘It would take time to develop the clinical experience with the test to weigh it against other biomarkers like hippocampal volume’ ^B^  ‘I might have to be more cautious in applying it initially, especially where there is not a convincing clinical history’ ^B^ |
| Knowledge and awareness | 1 | 1 | 0 | | Knowledge | ‘Need more in-depth knowledge on the biomarkers’ ^E^  ‘Lack of awareness’ ^B^ |

**Appendix 8.** **Summary of Open-text Survey Response Qualitative Findings Mapped to the TDF domains and Barriers and Enablers for AD** **BBM Testing.**

^a^ Statements marked with an ^E^ indicate enablers, with a ^B^ indicate barriers, and with an ^M^ indicated mixed enabler/barrier to the use of AD BBMs

Acronyms: ^1^Rxs: Treatments; ^2^MCI: Mild Cognitive Impairment; ^3^spec/sens: Specificity/Sensitivity; ^4^NHS: National Health Service; ^5^LP: Lumbar Puncture; ^6^FDG-PET: Fluorodeoxyglucose Positron Emission Tomography

|  | **Dependent Variable: Intention** | | | |
| --- | --- | --- | --- | --- |
| **TDF Domain** | **B** | **95% CI** | **Standard Error** | **P-Value** |
| Intercept | -2.70 | -4.36 to -1.04 | 0.84 | 0.002 ** |
| Knowledge | -0.04 | -0.19 to 0.11 | 0.07 | 0.587 |
| Memory, attention & decision processes | 0.44 | 0.20 to 0.68 | 0.12 | <0.001 *** |
| Skills | 0.03 | -0.20 to 0.25 | 0.12 | 0.827 |
| Behavioural regulation | 0.15 | -0.10 to 0.41 | 0.13 | 0.227 |
| Social/professional role & identity | 0.19 | -0.12 to 0.51 | 0.16 | 0.229 |
| Beliefs about capabilities | 0.21 | -0.00 to 0.43 | 0.11 | 0.055 |
| Optimism | -0.31 | -0.58 to -0.04 | 0.14 | 0.027* |
| Beliefs about consequences | 0.45 | 0.11 to 0.78 | 0.17 | 0.010** |
| Goals | 0.08 | -0.12 to 0.27 | 0.10 | 0.430 |
| Reinforcement | 0.01 | -0.26 to 0.27 | 0.13 | 0.962 |
| Emotion | -0.33 | -0.60 to -0.06 | 0.14 | 0.018* |
| Environmental context & resources | 0.11 | -0.03 to 0.25 | 0.07 | 0.127 |
| Social influences | 0.24 | 0.04 to 0.44 | 0.10 | 0.020* |
| **Observations** | **Value** | | | |
| R2 | 0.43 | | | |
| Adjusted R2 | 0.38 | | | |
| Residual Std. Error | 0.72 | | | |
| F Statistic | 9.13 | | | |
| * p value map | 0 ‘***’ 0.001 ‘**’ 0.01 ‘*’ 0.05 | | | |

**Appendix 9. Linear regression analysis examining the association between TDF domains and intention.**

|  | **Dependent Variable: Intention** | | | |
| --- | --- | --- | --- | --- |
| **TDF Domain** | **B** | **95% CI** | **Standard Error** | **P-Value** |
| Intercept | -2.78 | -4.48 to -1.08 | 0.86 | 0.002 ** |
| Knowledge | -0.04 | -0.19 to 0.11 | 0.08 | 0.636 |
| Memory, attention & decision processes | 0.43 | 0.19 to 0.67 | 0.12 | 0.001 *** |
| Skills | 0.02 | -0.21 to 0.25 | 0.12 | 0.842 |
| Behavioural regulation | 0.15 | -0.11 to 0.41 | 0.13 | 0.246 |
| Social/professional role & identity | 0.20 | -0.12 to 0.52 | 0.16 | 0.214 |
| Beliefs about capabilities | 0.21 | -0.01 to 0.43 | 0.11 | 0.064 |
| Optimism | -0.31 | -0.59 to -0.03 | 0.14 | 0.031* |
| Beliefs about consequences | 0.47 | 0.12 to 0.81 | 0.18 | 0.009** |
| Goals | 0.09 | -0.11 to 0.29 | 0.10 | 0.372 |
| Reinforcement | 0.00 | -0.28 to 0.28 | 0.14 | 0.998 |
| Emotion | -0.32 | -0.60 to -0.05 | 0.14 | 0.021* |
| Environmental context & resources | 0.11 | -0.03 to 0.26 | 0.07 | 0.117 |
| Social influences | 0.23 | 0.02 to 0.43 | 0.10 | 0.031* |
| **Observations** | **Value** | | | |
| R2 | 0.43 | | | |
| Adjusted R2 | 0.37 | | | |
| Residual Std. Error | 0.73 | | | |
| F Statistic | 6.89 | | | |
| * p value map | 0 ‘***’ 0.001 ‘**’ 0.01 ‘*’ 0.05 | | | |

**Appendix 10. Sensitivity analysis: Multiple linear regression predicting intention, additionally adjusted for consultant status and geographic region**

|  | **Mean Scores** |  | **% agreement** |  |  | p-value (Fisher’s exact) |
| --- | --- | --- | --- | --- | --- | --- |
| **Intervention** | Mean | SD | All | Memory service clinicians | Other Clinicians |  |
| **Further education** |  |  |  |  |  |  |
| Educational materials e.g. handouts | 4.3 | 0.7 | 92.4 | 91.6 | 93.9 | 0.8 |
| Workshops or seminars | 4.4 | 0.6 | 96.5 | 95.3 | 98.5 | 0.4 |
| **Training** |  |  |  |  |  |  |
| Online modules or e-learning courses | 4.3 | 0.8 | 92.4 | 94.4 | 89.2 | 0.2 |
| Incorporate blood biomarkers for Alzheimer's disease into the professional training (ST) curricula | 4.4 | 0.6 | 96.5 | 97.2 | 95.4 | 0.7 |
| **Guidelines** |  |  |  |  |  |  |
| National Appropriate Use Guidelines | 4.5 | 0.6 | 97.7 | 97.2 | 98.5 | 1.0 |
| Guidelines on How To Interpret The Result | 4.6 | 0.5 | 99.4 | 99.1 | 100.0 | 1.0 |
| Guidelines on how to incorporate the result into the diagnostic pathway | 4.5 | 0.6 | 97.1 | 95.3 | 100.0 | 0.2 |
| **Persuasion** |  |  |  |  |  |  |
| Promotion of adherence to guidelines through audit and feedback | 4.2 | 0.7 | 87.8 | 90.7 | 83.1 | 0.2 |
| **Incentives** |  |  |  |  |  |  |
| Meeting Department of Health targets for dementia subtype diagnosis rates | 3.4 | 1.2 | 52.3 | 49.5 | 56.9 | 0.4 |
| **Modelling** |  |  |  |  |  |  |
| Case examples from key opinion leaders (e.g. well-known researchers in the field of blood biomarkers for Alzheimer's disease) | 3.9 | 0.9 | 77.3 | 75.7 | 80.0 | 0.6 |
| Case examples from memory service colleagues who have successfully implemented the blood biomarker test | 4.1 | 0.7 | 87.2 | 88.8 | 84.6 | 0.5 |
| **Enablement** |  |  |  |  |  |  |
| Online forums or peer groups to share experiences and problem solving strategies | 3.8 | 0.9 | 70.4 | 70.1 | 70.8 | 1.0 |
| **Changes Needed Within the Service Environment** |  |  |  |  |  |  |
| Access to phlebotomy equipment | 4.4 | 0.6 | 94.2 | 95.3 | 92.3 | 0.5 |
| Access to trained phlebotomy staff | 4.4 | 0.7 | 94.8 | 96.3 | 92.3 | 0.3 |
| Clinic Room Access | 4.4 | 0.6 | 96.5 | 95.3 | 98.5 | 0.4 |
| Blood Specimen Transportation | 4.4 | 0.6 | 95.4 | 96.3 | 93.9 | 0.5 |
| Access to a laboratory for analysis | 4.4 | 0.6 | 95.9 | 96.3 | 95.4 | 1.0 |
| Access to a results system | 4.4 | 0.6 | 95.9 | 95.3 | 96.9 | 0.7 |

**Appendix 11. Mean scores and percentage agreement with belief statements representing intervention functions for AD BBM use by service (memory service vs other service)**

| **Intervention Function** | **Statement** |
| --- | --- |
| Education | Guidance on where to use the test appropriately will be vital |
|  | Patient and carer focussed information would be essential especially given false positive and false negative rates |
|  | Memory nurses may face difficulties due to lack of basic science and knowledge training and the fact that manh consultants do not see patients first hand but rely on nursing assessments, those who are seen at home and can not access the clinic mah nit be able to benefit from bio markers. |
| Training | Memory nurses may face difficulties due to lack of basic science and knowledge training and the fact that manh consultants do not see patients first hand but rely on nursing assessments, those who are seen at home and can not access the clinic mah nit be able to benefit from bio markers. |
| Environmental restructuring | Our memory clinic has been told it needs to vacate its premises in the next three months. With this sort of uncertainty, it's very hard to imagine new improvements such as a phlebotomy room etc |
|  | Could the sample not be drawn at patient's convenience in a standard hospital phlebotomy department? |
|  | Currently there is limited phlebotomy service and this could impose a huge demand on service that needs to be ironed out before implementation |
|  | We would not necessarily need to set up taking the blood samples in our memory clinics as we use the phlebotomy services at our 2 local general hospitals |
|  | Effective arrangements will be needed so that the specimen is analysed within the timeframe advised by the manufacturer |
|  | Memory nurses may face difficulties due to lack of basic science and knowledge training and the fact that manh consultants do not see patients first hand but rely on nursing assessments, those who are seen at home and can not access the clinic mah nit be able to benefit from bio markers. |
| Enablement | Funding is a key issue |
|  | Effective arrangements will be needed so that the specimen is analysed within the timeframe advised by the manufacturer |
| Coercion | It must be stressed that incorporation of this test in routine practice will not necessarily increase treatment efficacy. |
|  | The survey is too long and repetitive. The point is - if there is a reliable test, provision should be made to administer it when it’s clinically indicated. Current lack of clinical provision is irrelevant. It’s also very important to recognise that the vast majority of diagnoses are straightforward and based on symptomatology - there is no role for a test with 80% spec/sens in the vast majority of cases (note that’s 80% compared with gold standard of PET, which is hardly a gold standard itself - the figures ought to be compared against histopathological findings, not another low-specification investigation). |
| Incentivisation | appealing. Obviously clinical assessment would trump any test so the test would then be â€˜ gold standard to support & diagnosis and different type.1 |
|  | A Blood investigation for diagnoatic purposes in Alzheimer's disease a welcome development; however this should not take the place of current robust clinical and occupational diagnosis of the disease. The role of the Blood Test in sensitively providing a Mild Cognitive Impairment exclusion is also a welcome benefit. |
| Persuasion | Please do not use “opinion leaders” and “experts” as they are often drug company funded biased and very biomedically orientated. I change my practice on the basis of good scientific evidence. |

**Appendix 12. Summary of free text responses for strategies to improve AD BBM Testing mapped to the intervention functions in the Behaviour Change Wheel.**

| **Domain** | **Mean Scores** |  | **% agreement** |  |  | **p-value (Fisher’s exact)** |
| --- | --- | --- | --- | --- | --- | --- |
| **Knowledge** | Mean | SD | All | Memory Service Clinicians | Other Clinicians |  |
| I am familiar with the evidence base supporting the use of blood biomarkers for Alzheimer’s disease | 3.2 | 1.1 | 47.1 | 52.3 | 38.5 | 0.1 |
| I have a good understanding of what blood biomarkers for Alzheimer’s disease measure | 3.4 | 0.9 | 55.2 | 57.9 | 50.8 | 0.4 |
| I know the currently published appropriate context of use recommendations to request a blood biomarker for Alzheimer’s disease | 2.8 | 1.0 | 26.7 | 29.0 | 23.1 | 0.5 |
| **Memory, attention, and decision processes** |  |  |  |  |  |  |
| I would bear in mind using a blood biomarker for Alzheimer’s disease as part of my routine clinical practice | 3.8 | 1.0 | 71.5 | 77.6 | 61.5 | 0.0 |
| Using a blood biomarker result for Alzheimer’s disease would strongly inform my clinical decision making around diagnosis | 3.8 | 0.9 | 68.0 | 72.0 | 61.5 | 0.2 |
| Using a blood biomarker for Alzheimer's disease would require me to expend significantly more effort in making a decision than current practice | 2.62 | 1.0 | 20.9 | 20.6 | 21.5 | 1.0 |
| **Cognitive and Interpersonal Skills** |  |  |  |  |  |  |
| It would require technical skill to take a blood sample for a biomarker test for Alzheimer’s disease | 2.6 | 1.1 | 26.2 | 24.3 | 29.2 | 0.5 |
| I feel confident I could communicate a blood biomarker for Alzheimer’s disease result to colleagues in the team | 3.8 | 0.9 | 72.1 | 74.8 | 67.7 | 0.4 |
| I feel confident I could communicate a blood biomarker for Alzheimer’s disease result to patients and their families | 3.7 | 0.9 | 71.5 | 72.0 | 70.8 | 0.9 |
| **Behavioural Regulation** |  |  |  |  |  |  |
| I would want all patients presenting to our clinic for investigation of possible dementia to have this test | 2.8 | 1.1 | 30.8 | 27.1 | 36.9 | 0.2 |
| I would work with my colleagues in our service to develop local policy for use of the test | 4.16 | 0.66 | 88.4 | 92.5 | 81.5 | 0.0 |
| I would be willing to compare my use of this blood biomarker test with local colleagues | 4.3 | 0.6 | 95.3 | 98.1 | 90.8 | 0.1 |
| **Social/professional role and identify** |  |  |  |  |  |  |
| It is my responsibility to use the latest advancements in diagnostic technology when they become available for my patients | 4.1 | 0.8 | 82.0 | 84.1 | 78.5 | 0.4 |
| Using a blood biomarker test for Alzheimer’s disease would align with best practice for dementia diagnosis | 3.8 | 0.8 | 70.3 | 73.8 | 64.6 | 0.2 |
| I have ethical concerns to using a blood biomarker test for Alzheimer’s disease in clinical practice (specifically not genetic blood tests such as APOE status) | 2.4 | 0.9 | 14.5 | 15.0 | 13.8 | 1.0 |
| A delay in time between MHRA (Medicines and Healthcare products Regulatory Agency) approval and NICE guidance for a blood biomarker test for Alzheimer's disease would stop me ordering the test | 3.3 | 1.0 | 48.3 | 43.9 | 55.4 | 0.2 |
| Clinicians working in old age psychiatry should use a blood biomarker test to improve diagnoses in Alzheimer’s disease | 3.8 | 0.8 | 70.9 | 76.6 | 61.5 | 0.0 |
| **Belief about capabilities** |  |  |  |  |  |  |
| I have confidence in my ability to use a blood biomarker for Alzheimer’s disease test in my diagnostic practice | 3.4 | 1.0 | 53.5 | 57.9 | 46.2 | 0.2 |
| I would feel confident my allied health professional colleagues would be able to use a blood biomarker for Alzheimer’s disease test in their diagnostic practice | 2.5 | 1.0 | 16.9 | 16.8 | 16.9 | 1.0 |
| I feel confident that I could interpret the result of a blood biomarker for Alzheimer’s disease | 3.5 | 1.0 | 57.0 | 60.7 | 50.8 | 0.2 |
| **Optimism** |  |  |  |  |  |  |
| I believe a blood biomarker for Alzheimer’s disease will have high accuracy for detecting Alzheimer’s pathology in the brain | 3.5 | 0.8 | 52.9 | 57.9 | 44.6 | 0.1 |
| I believe a blood biomarker for Alzheimer’s disease would improve how Alzheimer’s dementia is diagnosed | 4.0 | 0.8 | 84.3 | 86.9 | 80.0 | 0.3 |
| I do not believe a blood biomarker for Alzheimer’s disease would improve how Alzheimer’s dementia is treated | 2.4 | 1.1 | 18.0 | 15.9 | 21.5 | 0.4 |
| **Belief about consequences** |  |  |  |  |  |  |
| I will have access to more helpful information to guide diagnosis | 4.0 | 0.6 | 87.8 | 86.9 | 89.2 | 0.8 |
| It may reassure the patient/family that the diagnosis is reliable | 4.0 | 0.6 | 86.6 | 86.9 | 86.2 | 1.0 |
| It would help me facilitate access to medication licensed for Alzheimer’s disease | 3.6 | 1.0 | 65.1 | 63.6 | 67.7 | 0.6 |
| It will help to establish the diagnosis of Alzheimer’s disease earlier | 4.0 | 0.7 | 82.6 | 81.3 | 84.6 | 0.7 |
| The test result may have indeterminant (not black and white) values | 4.0 | 0.7 | 82.6 | 86.9 | 75.4 | 0.1 |
| The result may conflict with a diagnosis suggested by the clinical presentation | 3.0 | 0.7 | 80.2 | 84.1 | 73.8 | 0.1 |
| The result may conflict with the results of other investigations (e.g. imaging) | 3.7 | 0.7 | 72.1 | 72.0 | 72.3 | 1.0 |
| In a busy clinic the additional time and waiting for a result associated with the blood test could prevent it’s use | 2.7 | 1.0 | 26.7 | 27.1 | 26.2 | 1.0 |
| **Goals** |  |  |  |  |  |  |
| My personal target is to reduce the number of patients who are diagnosed with MCI | 3.0 | 1.0 | 34.3 | 35.5 | 32.3 | 0.7 |
| There are targets in my service related to increasing the proportion of patients with a pathological diagnosis | 2.7 | 1.1 | 23.3 | 27.1 | 16.9 | 0.1 |
| It would be a priority for me to incorporate a blood biomarker test for Alzheimer’s disease in my practice, relative to currently available investigations (e.g. brain imaging) | 3.4 | 1.0 | 54.7 | 57.0 | 50.8 | 0.4 |
| **Reinforcement** |  |  |  |  |  |  |
| Having access to a blood biomarker test for Alzheimer’s disease will make me more confident in my diagnostic skill | 3.7 | 0.9 | 70.3 | 72.0 | 67.7 | 0.6 |
| Commissioners of services are more likely to fund our service if we are using a blood biomarker test for Alzheimer’s disease as part of our assessment protocol | 3.1 | 0.9 | 27.9 | 25.2 | 32.3 | 0.4 |
| I would avoid using the blood biomarker test for Alzheimer's disease because a positive result could distress my patients | 1.8 | 0.7 | 1.2 | 0.9 | 1.5 | 1.0 |
| **Emotion** |  |  |  |  |  |  |
| I feel positive about using a blood biomarker test for Alzheimer’s disease in clinical practice | 3.9 | 0.8 | 76.7 | 76.6 | 76.9 | 1.0 |
| I feel frustrated about having to change what I do currently in the diagnostic assessment of Alzheimer’s disease | 1.9 | 0.7 | 3.5 | 2.8 | 4.6 | 0.7 |
| I feel threatened that the result of a blood biomarker test for Alzheimer’s disease may replace my expertise and skills | 1.8 | 0.8 | 4.1 | 1.9 | 7.7 | 0.1 |
| **Environmental- Context and resources** |  |  |  |  |  |  |
| I have access to an appropriate space (e.g. clinic room) to use a blood biomarker test for Alzheimer’s disease in clinical practice | 3.3 | 1.2 | 55.8 | 50.5 | 64.6 | 0.1 |
| I have access to basic blood test equipment to use a blood biomarker test for Alzheimer’s disease in clinical practice | 3.2 | 1.2 | 53.5 | 45.8 | 66.2 | 0.0 |
| There are available trained staff in phlebotomy to use a blood biomarker test for Alzheimer’s disease in clinical practice | 3.1 | 1.2 | 45.9 | 41.1 | 53.8 | 0.1 |
| I have access to blood sample transportation to use a blood biomarker test for Alzheimer’s disease in clinical practice | 3.1 | 1.2 | 45.9 | 39.3 | 56.9 | 0.0 |
| I have access to a laboratory to analyse any blood samples I may request in clinical practice | 3.2 | 1.1 | 48.8 | 48.6 | 49.2 | 1.0 |
| I have enough time to incorporate a blood biomarker test for Alzheimer’s disease in clinical practice | 3.5 | 0.9 | 57.0 | 54.2 | 61.5 | 0.4 |
| **Social Influence** |  |  |  |  |  |  |
| Most of my colleagues within my professional discipline would think that using a blood biomarker test for Alzheimer’s disease is a good idea | 3.7 | 0.8 | 65.1 | 72.0 | 53.8 | 0.0 |
| Most of my patients would think that using a blood biomarker test for Alzheimer’s disease is a good idea | 3.9 | 0.7 | 71.5 | 74.8 | 66.2 | 0.2 |
| Most of my patients’ families would think that using a blood biomarker test for Alzheimer’s disease is a good idea | 3.9 | 0.7 | 75.6 | 79.4 | 69.2 | 0.1 |
| **Intentions (-3 to 3 Likert scale)** |  |  |  |  |  |  |
| I intend to use a blood biomarker test for Alzheimer’s disease in clinical practice if they are approved for use | 2.1 | 1.0 | 93.0 | 92.5 | 93.8 | 1.0 |
| I want to use a blood biomarker test in clinical practice if it is approved for use | 2.1 | 1.0 | 93.6 | 92.5 | 95.4 | 0.5 |
| I believe I will be able to use a blood biomarker test in clinical practice if it is approved for use | 1.8 | 1.1 | 88.4 | 86.9 | 90.8 | 0.6 |

**Appendix 13. Mean scores and percentage agreement with belief statements representing barriers and enablers to AD BBM use by service (memory service vs other service)**

| **Domain** | **Mean Scores** |  | **% agreement** |  |  | **p-value (Fisher’s exact)** |
| --- | --- | --- | --- | --- | --- | --- |
| **Knowledge** | Mean | SD | All | Academic | Non-academic |  |
| I am familiar with the evidence base supporting the use of blood biomarkers for Alzheimer’s disease | 3.2 | 1.1 | 47.1 | 50.0 | 43.0 | 0.4 |
| I have a good understanding of what blood biomarkers for Alzheimer’s disease measure | 3.4 | 0.9 | 55.2 | 60.7 | 48.8 | 0.1 |
| I know the currently published appropriate context of use recommendations to request a blood biomarker for Alzheimer’s disease | 2.8 | 1.0 | 26.7 | 28.6 | 24.4 | 0.6 |
| **Memory, attention, and decision processes** |  |  |  |  |  |  |
| I would bear in mind using a blood biomarker for Alzheimer’s disease as part of my routine clinical practice | 3.8 | 1.0 | 71.5 | 77.4 | 65.1 | 0.1 |
| Using a blood biomarker result for Alzheimer’s disease would strongly inform my clinical decision making around diagnosis | 3.8 | 0.9 | 68.0 | 67.9 | 68.6 | 1.0 |
| Using a blood biomarker for Alzheimer's disease would require me to expend significantly more effort in making a decision than current practice | 2.62 | 1.0 | 20.9 | 25.0 | 16.3 | 0.2 |
| **Cognitive and Interpersonal Skills** |  |  |  |  |  |  |
| It would require technical skill to take a blood sample for a biomarker test for Alzheimer’s disease | 2.6 | 1.1 | 26.2 | 22.6 | 29.1 | 0.4 |
| I feel confident I could communicate a blood biomarker for Alzheimer’s disease result to colleagues in the team | 3.8 | 0.9 | 72.1 | 79.8 | 65.1 | 0.04 |
| I feel confident I could communicate a blood biomarker for Alzheimer’s disease result to patients and their families | 3.7 | 0.9 | 71.5 | 78.6 | 65.1 | 0.06 |
| **Behavioural Regulation** |  |  |  |  |  |  |
| I would want all patients presenting to our clinic for investigation of possible dementia to have this test | 2.8 | 1.1 | 30.8 | 32.1 | 30.2 | 0.9 |
| I would work with my colleagues in our service to develop local policy for use of the test | 4.16 | 0.66 | 88.4 | 84.5 | 81.9 | 0.2 |
| I would be willing to compare my use of this blood biomarker test with local colleagues | 4.3 | 0.6 | 95.3 | 97.6 | 93.0 | 0.3 |
| **Social/professional role and identify** |  |  |  |  |  |  |
| It is my responsibility to use the latest advancements in diagnostic technology when they become available for my patients | 4.1 | 0.8 | 82.0 | 82.1 | 81.4 | 1.0 |
| Using a blood biomarker test for Alzheimer’s disease would align with best practice for dementia diagnosis | 3.8 | 0.8 | 70.3 | 75.0 | 67.4 | 0.3 |
| I have ethical concerns to using a blood biomarker test for Alzheimer’s disease in clinical practice (specifically not genetic blood tests such as APOE status) | 2.4 | 0.9 | 14.5 | 13.1 | 15.1 | 0.8 |
| A delay in time between MHRA (Medicines and Healthcare products Regulatory Agency) approval and NICE guidance for a blood biomarker test for Alzheimer's disease would stop me ordering the test | 3.3 | 1.0 | 48.3 | 51.2 | 46.5 | 0.6 |
| Clinicians working in old age psychiatry should use a blood biomarker test to improve diagnoses in Alzheimer’s disease | 3.8 | 0.8 | 70.9 | 71.4 | 70.9 | 1.0 |
| **Belief about capabilities** |  |  |  |  |  |  |
| I have confidence in my ability to use a blood biomarker for Alzheimer’s disease test in my diagnostic practice | 3.4 | 1.0 | 50.0 | 57.0 | 46.2 | 0.4 |
| I would feel confident my allied health professional colleagues would be able to use a blood biomarker for Alzheimer’s disease test in their diagnostic practice | 2.5 | 1.0 | 17.9 | 16.3 | 16.9 | 0.8 |
| I feel confident that I could interpret the result of a blood biomarker for Alzheimer’s disease | 3.5 | 1.0 | 61.9 | 52.3 | 50.8 | 0.2 |
| **Optimism** |  |  |  |  |  |  |
| I believe a blood biomarker for Alzheimer’s disease will have high accuracy for detecting Alzheimer’s pathology in the brain | 3.5 | 0.8 | 52.9 | 57.1 | 50.0 | 0.4 |
| I believe a blood biomarker for Alzheimer’s disease would improve how Alzheimer’s dementia is diagnosed | 4.0 | 0.8 | 84.3 | 82.1 | 86.0 | 0.5 |
| I do not believe a blood biomarker for Alzheimer’s disease would improve how Alzheimer’s dementia is treated | 2.4 | 1.1 | 18.0 | 17.9 | 18.6 | 1.0 |
| **Belief about consequences** |  |  |  |  |  |  |
| I will have access to more helpful information to guide diagnosis | 4.0 | 0.6 | 87.8 | 86.9. | 88.4 | 0.8 |
| It may reassure the patient/family that the diagnosis is reliable | 4.0 | 0.6 | 86.6 | 89.3 | 83.7 | 0.4 |
| It would help me facilitate access to medication licensed for Alzheimer’s disease | 3.6 | 1.0 | 65.1 | 65.5 | 65.1 | 1.0 |
| It will help to establish the diagnosis of Alzheimer’s disease earlier | 4.0 | 0.7 | 82.6 | 82.1 | 82.6 | 1.0 |
| The test result may have indeterminant (not black and white) values | 4.0 | 0.7 | 82.6 | 82.1 | 82.6 | 1.0 |
| The result may conflict with a diagnosis suggested by the clinical presentation | 3.0 | 0.7 | 80.2 | 78.6 | 81.4 | 0.7 |
| The result may conflict with the results of other investigations (e.g. imaging) | 3.7 | 0.7 | 72.1 | 76.2 | 68.6 | 0.3 |
| In a busy clinic the additional time and waiting for a result associated with the blood test could prevent it’s use | 2.7 | 1.0 | 26.7 | 32.1 | 20.9 | 0.1 |
| **Goals** |  |  |  |  |  |  |
| My personal target is to reduce the number of patients who are diagnosed with MCI | 3.0 | 1.0 | 34.3 | 35.7 | 33.7 | 0.9 |
| There are targets in my service related to increasing the proportion of patients with a pathological diagnosis | 2.7 | 1.1 | 23.3 | 20.2 | 26.7 | 0.4 |
| It would be a priority for me to incorporate a blood biomarker test for Alzheimer’s disease in my practice, relative to currently available investigations (e.g. brain imaging) | 3.4 | 1.0 | 54.7 | 52.4 | 57.0 | 0.6 |
| **Reinforcement** |  |  |  |  |  |  |
| Having access to a blood biomarker test for Alzheimer’s disease will make me more confident in my diagnostic skill | 3.7 | 0.9 | 70.3 | 67.9 | 72.1 | 0.6 |
| Commissioners of services are more likely to fund our service if we are using a blood biomarker test for Alzheimer’s disease as part of our assessment protocol | 3.1 | 0.9 | 27.9 | 32.1 | 24.4 | 0.3 |
| I would avoid using the blood biomarker test for Alzheimer's disease because a positive result could distress my patients | 1.8 | 0.7 | 1.2 | 1.2 | 1.2 | 1.0 |
| **Emotion** |  |  |  |  |  |  |
| I feel positive about using a blood biomarker test for Alzheimer’s disease in clinical practice | 3.9 | 0.8 | 76.7 | 82.1 | 72.1 | 0.1 |
| I feel frustrated about having to change what I do currently in the diagnostic assessment of Alzheimer’s disease | 1.9 | 0.7 | 3.5 | 3.6 | 3.5 | 1.0 |
| I feel threatened that the result of a blood biomarker test for Alzheimer’s disease may replace my expertise and skills | 1.8 | 0.8 | 4.1 | 2.4 | 5.8 | 0.4 |
| **Environmental- Context and resources** |  |  |  |  |  |  |
| I have access to an appropriate space (e.g. clinic room) to use a blood biomarker test for Alzheimer’s disease in clinical practice | 3.3 | 1.2 | 55.8 | 54.8 | 57.0 | 0.9 |
| I have access to basic blood test equipment to use a blood biomarker test for Alzheimer’s disease in clinical practice | 3.2 | 1.2 | 53.5 | 56.0 | 52.3 | 0.6 |
| There are available trained staff in phlebotomy to use a blood biomarker test for Alzheimer’s disease in clinical practice | 3.1 | 1.2 | 45.9 | 51.2 | 41.9 | 0.3 |
| I have access to blood sample transportation to use a blood biomarker test for Alzheimer’s disease in clinical practice | 3.1 | 1.2 | 45.9 | 48.8 | 44.2 | 0.6 |
| I have access to a laboratory to analyse any blood samples I may request in clinical practice | 3.2 | 1.1 | 48.8 | 56.0 | 43.0 | 0.1 |
| I have enough time to incorporate a blood biomarker test for Alzheimer’s disease in clinical practice | 3.5 | 0.9 | 57.0 | 65.5 | 48.8 | 0.03 |
| **Social Influence** |  |  |  |  |  |  |
| Most of my colleagues within my professional discipline would think that using a blood biomarker test for Alzheimer’s disease is a good idea | 3.7 | 0.8 | 65.1 | 71.4 | 59.3 | 0.1 |
| Most of my patients would think that using a blood biomarker test for Alzheimer’s disease is a good idea | 3.9 | 0.7 | 71.5 | 71.4 | 70.9 | 1.0 |
| Most of my patients’ families would think that using a blood biomarker test for Alzheimer’s disease is a good idea | 3.9 | 0.7 | 75.6 | 73.3 | 73.3 | 0.6 |
| **Intentions (-3 to 3 Likert scale)** |  |  |  |  |  |  |
| I intend to use a blood biomarker test for Alzheimer’s disease in clinical practice if they are approved for use | 2.1 | 1.0 | 93.0 | 95.2 | 90.7 | 0.4 |
| I want to use a blood biomarker test in clinical practice if it is approved for use | 2.1 | 1.0 | 93.6 | 92.9 | 94.2 | 0.8 |
| I believe I will be able to use a blood biomarker test in clinical practice if it is approved for use | 1.8 | 1.1 | 88.4 | 90.5 | 86.0 | 0.5 |

**Appendix 14. Mean scores and percentage agreement with belief statements representing barriers and enablers to AD BBM use by service (academic vs non-academic centre)**
