# Supplementary material for: Factors influencing implementation of an Alzheimer’s disease blood test among UK old age psychiatrists: mixed-methods study using the theoretical domains framework
Source: Age Ageing. 2026 May 6;55(5):afag117. doi: 10.1093/ageing/afag117 (PMC13147441; doi:10.1093/ageing/afag117)
Supplement: Supplementary_materials_afag117 [file supplementary_materials_afag117.zip › Supplementary_materials_afag117_File003.docx]

**Factors Influencing Implementation of an Alzheimer’s Disease Blood Test among UK Old Age Psychiatrists: a Theoretical Domains Framework-based Mixed-methods Study**

**Appendices**

**Contents**

**p.1 Appendix 1 – Survey Questions**

**p.16 Appendix 3 – Focus Group Topic Guide**

**Study 1: Online Survey for NHS Clinicians**

**Survey Information**

As NHS Clinicians, most of us have only limited experience of using fluid biomarkers in dementia diagnosis. This survey is part of research to develop an educational tool to guide us through the best ways to use and interpret the results from a new blood biomarker test for Alzheimer’s disease (p-tau217) that is likely to be available in the NHS, initially as part of clinical trials.

This survey explores your views and practices on the use of Alzheimer’s blood biomarkers, challenges and opportunities in their use and what could be done to support you in their practical implementation.

It will take approximately 10 minutes to complete and your responses are fully anonymous. The survey is part of Dr x’s PhD fellowship, funded by Alzheimer’s Research UK. In recognition of your time involved in completing the survey you can be entered into a prize draw for a £100 gift voucher.

For further information please see full Participant Information Sheet here (hyperlink)

**Survey Consent**

**Consent Form Summary:**

Please indicate your informed consent to take part in this study.

By consenting you are confirming that you've read and understood the Participant Information Sheet and consent to your responses to be stored anonymously, reported in research publications and stored for future research. The full consent form (hyperlink) can be viewed in detail here.

I consent to completing this survey ☐.

**Background**

Recent technological advancements have enabled the detection of Alzheimer's disease (AD) pathology in the blood. Plasma p-tau217 has emerged as a highly effective blood biomarker for distinguishing cognitively unimpaired individuals from those with AD, differentiating AD from other forms of dementia (such as frontotemporal dementia), and predicting the progression from mild cognitive impairment (MCI) to AD dementia in research settings.

For the blood test to be used as a confirmatory tool in clinical practice, it is recommended that plasma biomarkers demonstrate performance equivalent to that of CSF tests, particularly with 90% sensitivity and specificity in detecting amyloid-PET pathological status. Even with high test accuracy, there is still a chance of false-positive or false-negative results. It is expected that 80% of patients will receive a clearly positive or negative result, with approximately 20% falling into an indeterminate range, depending on how cut-off values are defined.

**Section 1. Demographics**

We ask for some basic demographic information such as your job role and work region. This information will be used solely for the purpose of describing the data sample. Your responses will remain confidential and will be anonymized in any published results.

Job details

- Years working in Old Age Psychiatry:
  - 1-5, 6-10, 11-15, 16-20, 20+
- Have you received training in any specialties other than psychiatry?
  - Neurology
  - General medicine
  - Other
- Position:
  - Specialist Trainee
  - Consultant
  - Speciality and Associate Specialist (SAS) doctor
  - Clinical Nurse Specialist
  - Advanced Nurse Practitioner
  - Other:
    - If other, Please specify your current role?:
- UK Region:
  - Wales
  - Scotland
  - Northern Ireland
  - England
    - North East
    - North West
    - Yorkshire and The Humber
    - East Midlands, West Midlands
    - East of England
    - London
    - South East
    - South West
- Do you work in a trust with an academic centre?
  - Yes
  - No
- Service(s) where you work: (Multiple answers are accepted. Check all that apply)
  - Inpatient setting
  - CMHT
  - Memory Service
  - Liaison Service
  - Other:

**Section 2. Current Practice**

- How many patients do you evaluate for possible Alzheimer’s disease?
- More than five a week
- Between one and five a week
- Less than once a week
- Once a month or less
- What proportion of your practice involves patients with Alzheimer’s disease?

1. Less than 25%
2. About 25-50%
3. More than 50%

- Which of the following do you routinely use when diagnosing Alzheimer's disease? (Check all that apply)
- Clinical evaluation (history, examination and cognitive assessment e.g. MOCA/MMSE/ACE-III)
- Referral to Neuropsychologist for neuropsychological assessment
- Neuroimaging: MRI
- Neuroimaging: CT
- Neuroimaging: Amyloid PET
- Neuroimaging: DAT scan
- CSF
- Other (please specify)
- In what % of patients do you order specialist amyloid biomarker investigations e.g. Lumbar Puncture/ Amyloid-PET
- 0%
- <10%
- 10-25%
- 25-50%
- >50%

• Which of the following would be reasons you would order specialist amyloid biomarker investigations?

1. To increase certainty of a diagnosis of Alzheimer’s disease

2. When the patient has a young onset (for example <65 years)

3. When the clinical presentation is atypical

4. To investigate the underlying aetiology of Mild Cognitive Impairment or dementia

5. Other: please specify

- What % of patients do you refer on to colleagues in Neurology for their specialist help in establishing a diagnosis?
- 0%
- <5%
- 5-10%
- >10%
- Have you ever ordered an Alzheimer’s disease CSF biomarkers or Amyloid PET in clinical practice?
  - Response: 1. Yes 2. No 3. Unsure

**Section 3.**

We are interested in learning more about the challenges and opportunities that you anticipate would be important when using a new blood biomarker for diagnosing Alzheimer’s disease.

Please rate the extent to which you agree with the following statements:

|  | Strongly disagree | Disagree | Neither agree or disagree | Agree | Strongly Agree |
| --- | --- | --- | --- | --- | --- |
| I am familiar with the evidence base supporting the use of blood biomarkers for Alzheimer’s disease |  |  |  |  |  |
| I have a good understanding of what blood biomarkers for Alzheimer’s disease measure |  |  |  |  |  |
| I know the currently published appropriate context of use recommendations to request a blood biomarker for Alzheimer’s disease |  |  |  |  |  |

When a blood biomarker for Alzheimer’s disease becomes available:

|  | Strongly disagree | Disagree | Neither agree or disagree | Agree | Strongly Agree |
| --- | --- | --- | --- | --- | --- |
| I would bear in mind using a blood biomarker for Alzheimer’s disease as part of my routine clinical practice |  |  |  |  |  |
| Using a blood biomarker result for Alzheimer’s disease would strongly inform my clinical decision making around diagnosis |  |  |  |  |  |
| Using a blood biomarker for Alzheimer's disease would require me to expend significantly more effort in making a decision than current practice |  |  |  |  |  |

1. When a blood biomarker test for Alzheimer’s disease becomes available, please rate the extent to which you agree with the following statements:

|  | Strongly disagree | Disagree | Neither agree or disagree | Agree | Strongly Agree |
| --- | --- | --- | --- | --- | --- |
| It would require technical skill to take a blood sample for a biomarker test for Alzheimer’s disease |  |  |  |  |  |
| I feel confident I could communicate a blood biomarker for Alzheimer’s disease result to colleagues in the team |  |  |  |  |  |
| I feel confident I could communicate a blood biomarker for Alzheimer’s disease result to patients and their families |  |  |  |  |  |

1. Please rate the extent to which you agree with the following statements:

|  | Strongly disagree | Disagree | Neither agree or disagree | Agree | Strongly Agree |
| --- | --- | --- | --- | --- | --- |
| I would want all patients presenting to our clinic for investigation of possible dementia to have this test |  |  |  |  |  |
| I would work with my colleagues in our service to develop local policy for use of the test |  |  |  |  |  |
| I would be willing to compare my use of this blood biomarker test with local colleagues |  |  |  |  |  |

1. Please rate the extent to which you agree with the following statements:

|  | Strongly disagree | Disagree | Neither agree or disagree | Agree | Strongly Agree |
| --- | --- | --- | --- | --- | --- |
| It is my responsibility to use the latest advancements in diagnostic technology when they become available for my patients |  |  |  |  |  |
| Using a blood biomarker test for Alzheimer’s disease would align with best practice for dementia diagnosis |  |  |  |  |  |
| I have ethical concerns to using a blood biomarker test for Alzheimer’s disease in clinical practice (specifically not genetic blood tests such as APOE status) |  |  |  |  |  |
| A delay in time between MHRA (Medicines and Healthcare products Regulatory Agency) approval and NICE guidance for a blood biomarker test for Alzheimer's disease would stop me ordering the test |  |  |  |  |  |
| Old age psychiatrists should use a blood biomarker test to improve diagnoses in Alzheimer’s disease |  |  |  |  |  |

1. Please rate the extent to which you agree with the following statements:

|  | Strongly disagree | Disagree | Neither agree or disagree | Agree | Strongly Agree |
| --- | --- | --- | --- | --- | --- |
| I have confidence in my ability to use a blood biomarker for Alzheimer’s disease test in my diagnostic practice |  |  |  |  |  |
| I would feel confident my allied health professional colleagues would be able to use a blood biomarker for Alzheimer’s disease test in their diagnostic practice |  |  |  |  |  |
| I feel confident that I could interpret the result of a blood biomarker for Alzheimer’s disease |  |  |  |  |  |

1. Please rate the extent to which you agree with the following statements:

|  | Strongly disagree | Disagree | Neither agree or disagree | Agree | Strongly Agree |
| --- | --- | --- | --- | --- | --- |
| I believe a blood biomarker for Alzheimer’s disease will have high accuracy for detecting Alzheimer’s pathology in the brain |  |  |  |  |  |
| I believe a blood biomarker for Alzheimer’s disease would improve how Alzheimer’s dementia is diagnosed |  |  |  |  |  |
| I believe a blood biomarker for Alzheimer’s disease would improve how Alzheimer’s dementia is treated |  |  |  |  |  |


What do you think will be the benefits of using a blood biomarker test for Alzheimer’s disease in clinical practice?

|  | Strongly disagree | Disagree | Neither agree or disagree | Agree | Strongly Agree |
| --- | --- | --- | --- | --- | --- |
| I will have access to more helpful information to guide diagnosis |  |  |  |  |  |
| It may reassure the patient/family that the diagnosis is reliable |  |  |  |  |  |
| It would help me facilitate access to medication licensed for Alzheimer’s disease |  |  |  |  |  |
| It will help to establish the diagnosis of Alzheimer’s disease earlier |  |  |  |  |  |

What do you think will be the challenges of using a blood biomarker test for Alzheimer’s disease in clinical practice?

|  | Strongly disagree | Disagree | Neither agree or disagree | Agree | Strongly Agree |
| --- | --- | --- | --- | --- | --- |
| The test result may have indeterminant (not black and white) values |  |  |  |  |  |
| The result may conflict with a diagnosis suggested by the clinical presentation |  |  |  |  |  |
| The result may conflict with the results of other investigations (e.g. imaging) |  |  |  |  |  |
| In a busy clinic the additional time and waiting for a result associated with the blood test could prevent it’s use |  |  |  |  |  |

1. Please rate the extent to which you agree with the following statements:

|  | Strongly disagree | Disagree | Neither agree or disagree | Agree | Strongly Agree |
| --- | --- | --- | --- | --- | --- |
| My personal target is to reduce the number of patients who are diagnosed with MCI |  |  |  |  |  |
| It would be a priority for me to incorporate a blood biomarker test for Alzheimer’s disease in my practice, relative to currently available investigations (e.g. brain imaging) |  |  |  |  |  |
| There are targets in my service related to increasing the proportion of patients with a pathological diagnosis |  |  |  |  |  |

1. Please rate the extent to which you agree with the following statements:

|  | Strongly disagree | Disagree | Neither agree or disagree | Agree | Strongly Agree |
| --- | --- | --- | --- | --- | --- |
| Having access to a blood biomarker test for Alzheimer’s disease will make me more confident in my diagnostic skill |  |  |  |  |  |
| Commissioners of services are more likely to fund our service if we are using a blood biomarker test for Alzheimer’s disease as part of our assessment protocol |  |  |  |  |  |
| When I use a blood biomarker test for Alzheimer’s disease in clinical practice, I feel like I am making a difference |  |  |  |  |  |
| I would avoid using the blood biomarker test for Alzheimer's disease in case a positive result distressed my patient |  |  |  |  |  |

1. Please rate the extent to which you agree with the following statements:

|  | Strongly disagree | Disagree | Neither agree or disagree | Agree | Strongly Agree |
| --- | --- | --- | --- | --- | --- |
| I feel positive about using a blood biomarker test for Alzheimer’s disease in clinical practice |  |  |  |  |  |
| I have concerns about using a blood biomarker test for Alzheimer’s disease in clinical practice |  |  |  |  |  |
| I feel frustrated about having to change what I do currently in the diagnostic assessment of Alzheimer’s disease |  |  |  |  |  |
| I feel threatened that the result of a blood biomarker test for Alzheimer’s disease may replace my expertise and skills |  |  |  |  |  |

1. Please rate the extent to which you agree with the following statements:

|  | Strongly disagree | Disagree | Neither agree or disagree | Agree | Strongly Agree |
| --- | --- | --- | --- | --- | --- |
| I have access to an appropriate space (e.g. clinic room) to use a blood biomarker test for Alzheimer’s disease in clinical practice |  |  |  |  |  |
| I have access to basic blood test equipment to use a blood biomarker test for Alzheimer’s disease in clinical practice |  |  |  |  |  |
| There are available trained staff in phlebotomy to use a blood biomarker test for Alzheimer’s disease in clinical practice |  |  |  |  |  |
| I have access to blood sample transportation to use a blood biomarker test for Alzheimer’s disease in clinical practice |  |  |  |  |  |
| I have access to a laboratory to analyse any blood samples I may request in clinical practice |  |  |  |  |  |
| I have enough time to incorporate a blood biomarker test for Alzheimer’s disease in clinical practice |  |  |  |  |  |

1. Please rate the extent to which you agree with the following statements:

|  | Strongly disagree | Disagree | Neither agree or disagree | Agree | Strongly Agree |
| --- | --- | --- | --- | --- | --- |
| Most of my colleagues within my professional discipline would think that using a blood biomarker test for Alzheimer’s disease was a good idea |  |  |  |  |  |
| In my service, most of my colleagues within my multi-disciplinary team would think that using a blood biomarker test for Alzheimer’s disease was a good idea |  |  |  |  |  |
| Most of my patients would think that using a blood biomarker test for Alzheimer’s disease was a good idea |  |  |  |  |  |
| Most of my patients’ families would think that using a blood biomarker test for Alzheimer’s disease was a good idea |  |  |  |  |  |

Is there anything else you see as a challenge or an opportunity in using a blood biomarker test for Alzheimer’s disease?

Free text response:

**Section 4:**

A new biomarker test for Alzheimer’s disease is being developed. If this was to be integrated into practice, please rate how likely would you be to use in practice?

|  | completely disagree | -3 | -2 | -1 | 0 | +1 | +2 | +3 | completely Agree |
| --- | --- | --- | --- | --- | --- | --- | --- | --- | --- |
| I intend to use a blood biomarker test for Alzheimer’s disease in clinical practice if they are approved for use |  |  |  |  |  |  |  |  |  |
| I want to use a blood biomarker test in clinical practice if it is approved for use |  |  |  |  |  |  |  |  |  |
| I believe I will be able to use a blood biomarker test in clinical practice if it is approved for use |  |  |  |  |  |  |  |  |  |

**Section 5:**

In this section, we seek your input on potential strategies to address the challenges and opportunities identified in the use of the Alzheimer's disease blood biomarker test.

Please consider the following potential strategies and rate your agreement with how helpful each might be in supporting the uptake of the biomarker test.

Please rate your level of agreement with the following educational or training interventions:

|  | Strongly disagree | Disagree | Neither agree or disagree | Agree | Strongly Agree |
| --- | --- | --- | --- | --- | --- |
| Educational materials e.g. handouts |  |  |  |  |  |
| Workshops or seminars |  |  |  |  |  |
| Online modules or e-learning courses |  |  |  |  |  |
| Incorporate blood biomarkers for Alzheimer's disease into the professional training curricula |  |  |  |  |  |

Please rate your level of agreement with changes needed within the Service Environment

|  | Strongly disagree | Disagree | Neither agree or disagree | Agree | Strongly Agree |
| --- | --- | --- | --- | --- | --- |
| Phlebotomy equipment |  |  |  |  |  |
| Trained phlebotomy staff |  |  |  |  |  |
| Room access |  |  |  |  |  |
| Blood specimen transportation |  |  |  |  |  |
| Access to a Laboratory for analysis |  |  |  |  |  |
| Access to a results system |  |  |  |  |  |

Please rate your level of agreement on access to guidelines for the use of a blood biomarker test for Alzheimer's disease

|  | Strongly disagree | Disagree | Neither agree or disagree | Agree | Strongly Agree |
| --- | --- | --- | --- | --- | --- |
| National appropriate use guidelines |  |  |  |  |  |
| Interpretation of the result |  |  |  |  |  |
| Incorporation of the result into the diagnostic pathway |  |  |  |  |  |
| Promote adherence to guidelines through audit |  |  |  |  |  |

How much would the following be an incentive for the use of the blood biomarker in clinical practice

|  | Strongly disagree | Disagree | Neither agree or disagree | Agree | Strongly Agree |
| --- | --- | --- | --- | --- | --- |
| Meeting department of Health targets for dementia subtype diagnosis rates |  |  |  |  |  |

How much would the provision of case examples to support the use of the blood biomarker for Alzheimer's disease in clinical practice be helpful

|  | Strongly disagree | Disagree | Neither agree or disagree | Agree | Strongly Agree |
| --- | --- | --- | --- | --- | --- |
| Case examples from key opinion leaders (e.g. well-known researchers in the field of blood biomarkers for Alzheimer's disease) |  |  |  |  |  |
| Case examples from memory service colleagues who have successfully implemented the blood biomarker test |  |  |  |  |  |

How useful would you find the following methods to support the use of a blood biomarker for Alzheimer's disease in clinical practice

|  | Strongly disagree | Disagree | Neither agree or disagree | Agree | Strongly Agree |
| --- | --- | --- | --- | --- | --- |
| Online forums or peer groups to share experiences and problem solving strategies |  |  |  |  |  |

Please use the space below for any additional comments that have not been covered in the survey.

[Free text response]

**Thank You**

Thank you for participating in our survey.

**Consent to Participate in Further Research**

I consent to sharing my name and email address with the research team so I can be contacted about further research opportunities related to this study, which may include focus group discussions.

- Yes
- No

If Yes:

Name:­­­­ __________________________

Email Address: __________________________

**Consent to Share Email Address for Prize Draw**

I consent to sharing my email address for a chance to win £100 in a prize draw.

- Yes
- No

If Yes:

Email Address: __________________________

**Focus Group Guide**

So far, we have conducted a literature review and a survey to better understand the challenges and opportunities associated with using the new blood test for Alzheimer's disease in clinical practice. The literature review included qualitative studies reflecting perceptions of patients, caregivers, and health care professionals (HCPs) on Alzheimer’s Disease (AD) biomarker investigations. The survey, which was disseminated through the Royal College of Psychiatrists, Faculty of Old Age Psychiatry, focused on psychiatrists' perspectives.

I’d like to discuss the barriers (challenges) and enablers (opportunities) that were identified in these studies and hear your thoughts and insights. We will discuss each of these in turn.

The first set of key challenges identified were related to gaps in knowledge.

Some of the main barriers identified include:

• Understanding what the blood test is measuring and its limitations. For example, a positive result provides evidence of Alzheimer’s pathology, which is different from a clinical diagnosis of Alzheimer’s disease.

• Uncertainty about the appropriate use context and guidelines for testing, with specific concerns around testing of asymptomatic individuals.

• Challenges with understanding the diagnostic accuracy of the test and its validation, particularly in diverse populations, patients with medical comorbidities, or people >80 years old.

**Question:**

**“**Do these findings resonate with your experiences? Are there any additional knowledge-related challenges or opportunities that are important?”

Thank you for your insights. I’d now like to discuss factors related to skills in interpreting and communicating the results. Barriers to testing include:

• Confidence in how to integrate the biomarker test result within the clinical assessment and interpret the result.

- The challenge of how to interpret blood test results when they are indeterminate, or conflict with clinical impressions or imaging results.

• The way we communicate can lead to misunderstanding about the meaning of the test results. For instance, some patients interpreted a “positive amyloid PET scan result” as a favourable outcome, not understanding its full implications.

On the other hand, there are enablers:

• Visual tools and printouts have been found to be helpful for patients, caregivers, and HCPs as a means of communicating the results of the blood test more clearly.

**Question:**

"Do you foresee yourself facing similar challenges?"

Thank you, I’d like to move onto the next set of factors which relate to your beliefs on the consequences of testing.

• Some individuals believe testing can enable access to earlier diagnosis and treatment, or aid in the differential diagnosis.

• On the other hand, some express concern that the test may lead to disappointment if the results are inconclusive or do not provide definitive answers about diagnosis.

• Other concerns expressed relate to the value of the test in patients over the age of 80, or those with multiple comorbidities.

• There are also concerns raised regarding legal and ethical implications of the result, and the possible consequences of false positives and false negatives.

**Question:**

"What are your thoughts on this?"

Thank you, this is very useful. Let’s now consider the possible emotional impacts associated with testing. The testing process can be emotionally burdensome for both the patient and clinician.

**Question:**

Is this something that would concern you?

*Prompt: How does the prospect of using a blood biomarker test for Alzheimer’s disease in clinical practice make you feel?*

Thank you everyone. For this next part, I’d like us to consider how your professional role and identity as healthcare providers may also shape how you would use a biomarker test in practice. For instance, clinicians may place different weighting on involving patients actively in the decision-making process of whether to have a biomarker test. Some prefer a more collaborative approach, whereas others may take a more directive stance.

**Question:**

“How does this resonate with how you approach shared decision-making and your professional role in testing decisions?”

Thank you all for your participation. Are there any other issues that you can identify in relating to biomarker testing we have not discussed so far today?

**Interventions**

Finally, having considered barriers to testing, I would like to ask for your input on a potential intervention to address them.

“What do you think could be done to address some of the challenges we have discussed today?”

We are developing an e-learning module or tool to support clinicians in using this blood test.

- What barriers do you think should be addressed in this e-tool?
- What should be include in it?
- What would be feasible to request clinicians to complete as part of this e-tool?
- What design features would you want the e-learning tool to include, for instance what would make the e-tool more engaging or interesting to use?

*Prompt:* *What has been good about previous mandatory training e-modules? What has been less good about them?*

- How long would you be willing to spend on this tool e.g. for Continuous Professional Development (CPD)? What would motivate you to use it?
- We are considering including patient testimonials and perspectives on the use of this blood test during diagnostic assessments. What are your thoughts on this?

**Other**

Before we finish, is there anything you would like to mention or ask that we have not already covered?
